# Mapping Connectivity Damage in the Case of Phineas Gage

**DOI:** 10.1371/journal.pone.0037454

**Published:** 2012-05-16

**Authors:** John Darrell Van Horn, Andrei Irimia, Carinna M. Torgerson, Micah C. Chambers, Ron Kikinis, Arthur W. Toga

**Affiliations:** 1 Laboratory of Neuro Imaging (LONI), Department of Neurology, David Geffen School of Medicine, University of California Los Angeles, Los Angeles, California, United States of America; 2 Surgical Planning Laboratory, Department of Radiology, Brigham & Women's Hospital, Harvard Medical School, Boston, Massachusetts, United States of America; Indiana University, United States of America

## Abstract

White matter (WM) mapping of the human brain using neuroimaging techniques has gained considerable interest in the neuroscience community. Using diffusion weighted (DWI) and magnetic resonance imaging (MRI), WM fiber pathways between brain regions may be systematically assessed to make inferences concerning their role in normal brain function, influence on behavior, as well as concerning the consequences of network-level brain damage. In this paper, we investigate the detailed connectomics in a noted example of severe traumatic brain injury (TBI) which has proved important to and controversial in the history of neuroscience. We model the WM damage in the notable case of Phineas P. Gage, in whom a “tamping iron” was accidentally shot through his skull and brain, resulting in profound behavioral changes. The specific effects of this injury on Mr. Gage's WM connectivity have not previously been considered in detail. Using computed tomography (CT) image data of the Gage skull in conjunction with modern anatomical MRI and diffusion imaging data obtained in contemporary right handed male subjects (aged 25–36), we computationally simulate the passage of the iron through the skull on the basis of reported and observed skull fiducial landmarks and assess the extent of cortical gray matter (GM) and WM damage. Specifically, we find that while considerable damage was, indeed, localized to the left frontal cortex, the impact on measures of network connectedness between directly affected and other brain areas was profound, widespread, and a probable contributor to both the reported acute as well as long-term behavioral changes. Yet, while significantly affecting several likely network hubs, damage to Mr. Gage's WM network may not have been more severe than expected from that of a similarly sized “average” brain lesion. These results provide new insight into the remarkable brain injury experienced by this noteworthy patient.

## Introduction

The mapping of human brain connectivity through the use of modern neuroimaging methods has enjoyed considerable interest, examination, and application in recent years [1], [2]. Through the use of diffusion weighted (DWI) and magnetic resonance imaging (MRI), it is possible to systematically assess white matter (WM) fiber pathways between brain regions to measure fiber bundle properties, their influence on behavior and cognition, as well as the results of severe brain damage. The potential for using combined DWI/MRI methods to understand network-level alterations resulting from neurological insult is among their major research and clinical advantages.

**Figure 1:**
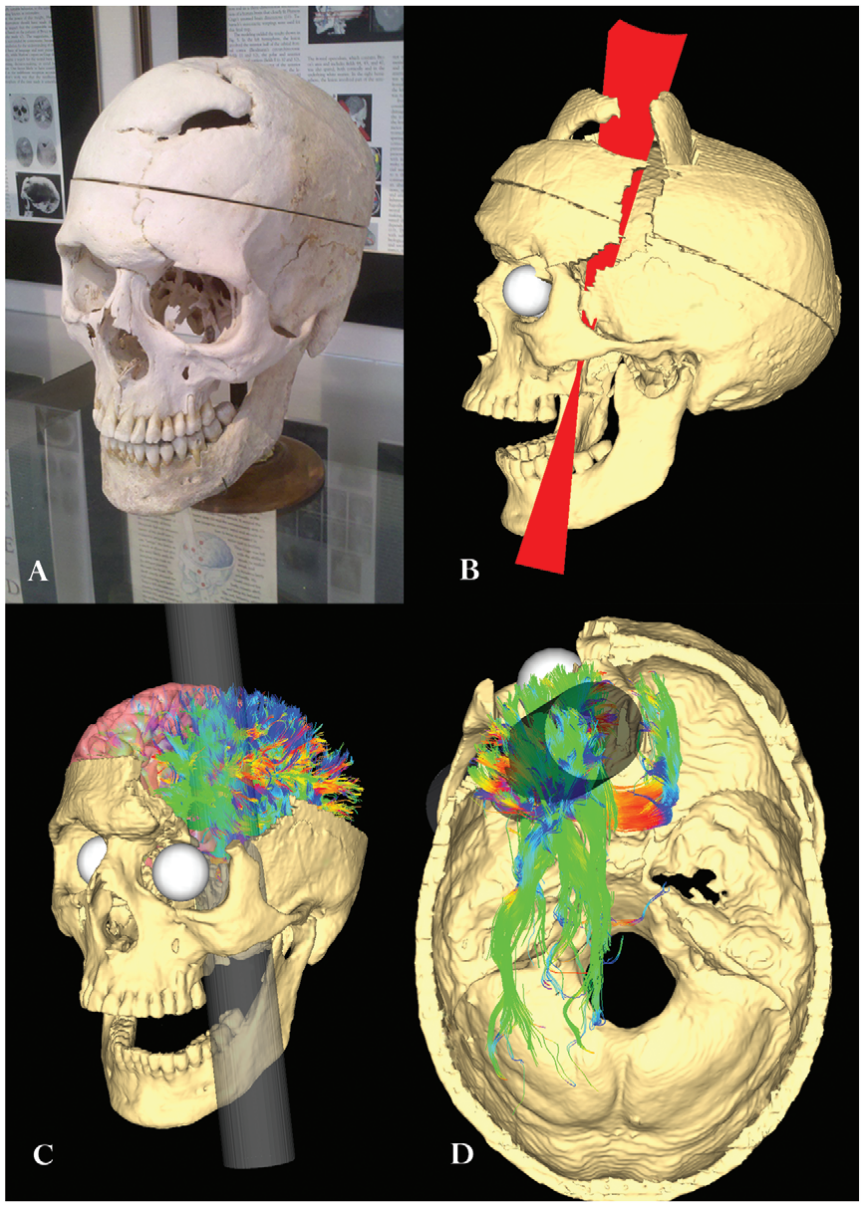
Modeling the path of the tamping iron through the Gage skull and its effects on white matter structure. a) The skull of Phineas Gage on display at the Warren Anatomical Museum at Harvard Medical School. b) CT image volumes were reconstructed, spatially aligned, and manual segmentation of the individual pieces of bone dislodged by the tamping iron (rod), top of the cranium, and mandible was performed. Surface meshes for each individual element of the skull were created. Based upon observations from previous examinations of the skull as well as upon the dimensions of the iron itself, fiducial constraint landmarks were digitally imposed and a set of possible rod trajectories were cast through the skull. This figure shows the set of possible rod trajectory centroids which satisfied each of the anatomical constraints. The trajectory nearest the mean trajectory was considered the true path of the rod and was used in all subsequent calculations. Additionally, voxels comprising the interior boundary and volume of the cranial vault were manually extracted and saved as a digital endocast of Mr. Gage's brain cavity. c) A rendering of the Gage skull with the best fit rod trajectory and example fiber pathways in the left hemisphere intersected by the rod. Graph theoretical metrics for assessing brain global network integration, segregation, and efficiency [92] were computed across each subject and averaged to measure the changes to topological, geometrical, and wiring cost properties. d) A view of the interior of the Gage skull showing the extent of fiber pathways intersected by the tamping iron in a sample subject (*i.e.* one having minimal spatial deformation to the Gage skull). The intersection and density of WM fibers between all possible pairs of GM parcellations was recorded, as was average fiber length and average fractional anisotropy (FA) integrated over each fiber.

In this paper, we investigate the detailed connectomics of a noted example of severe traumatic brain injury (TBI) which has proved important to and controversial in the history of neuroscience. Few cases in the history of the medical sciences have been so important, interpreted, and misconstrued, as the case of Phineas P. Gage [3], in whom a “tamping iron” was accidentally shot through his skull and brain, resulting in profound behavioral changes, and which contributed to his death 151 years ago. On September 13th, 1848, the 25-year old Phineas P. Gage was employed as a railroad construction supervisor near Cavendish, Vermont to blast and remove rock in preparation for the laying of the Rutland and Burlington Railroad. Having drilled a pilot hole into the rock and filling it partially with gunpowder, he instructed an assistant to pour sand into the hole atop the powder. Averting his attention for a moment to speak with his men, he apparently assumed the sand had been added. He then commenced dropping the end of a 110 cm long, 3.2 cm diameter iron rod into the hole in order to “tamp” down its contents. The 13 lb. iron struck the interior wall of the hole causing a spark to ignite the powder which, in turn, launched the pointed iron rod upwards, through the left cheek of Mr. Gage just under the zygomatic arch, passing behind his left eyeball, piercing his cranial vault under the left basal forebrain, passing through his brain, and then exiting the top and front of his skull near the sagittal suture. A large amount of brain tissue was expelled from the opening and the rod was found later “smeared with blood and brains”, washed in a stream, and, eventually, returned to him. After receiving treatment and care from Dr. John Martyn Harlow over subsequent weeks, Mr. Gage was able to recover sufficiently from his physical injuries and return to his family in nearby New Hampshire. However, reports of profound personality changes indicate that he was unable to return to his previous job and caused co-workers to comment that he was “no longer Gage.” Following several years of taking manual labor jobs and travelling throughout New England and eventually to Valparaiso, Chile, always in the company of “his iron”, he was reunited with his family in San Francisco whereupon Mr. Gage died on May 21, 1860, nearly 12 years after his injury – presumably due to the onset of seizures evidently originating from damage resulting from the tamping rod incident. Several years later, Dr. Harlow, upon learning of Gage's death, asked Gage's sister's family to exhume his body to retrieve his skull and rod for presentation to the Massachusetts Historical Society and deposition with Harvard Medical School where, to this day, it remains on display in the Warren Anatomical Museum in the Francis A. Countway Library of Medicine at Harvard Medical School (Fig. 1a).

The amount of damage to Mr. Gage's left frontal cortical grey matter (GM) with secondary damage to surrounding GM has been considered by several authors with reference to Gage's reported change in temperament, character, etc [4], [5], [6] (Table 1). With the aid of medical imaging technology, two previous published articles have sought to illustrate the impact of the rod on Mr. Gage's skull and brain. Most famously, Damasio *et al.* [7] illustrated that the putative extent of damage to the left frontal cortex would be commensurate with the disinhibition, failures to plan, memory deficiencies, and other symptoms noted in patients having frontal lobe injury. Ratiu *et al.* [8] sought to illustrate the trajectory of the tamping iron, characterize the pattern of skull damage, and explain potential brain damage using a single, example subject. However, while many authors have focused on the gross damage done by the iron to Gage's frontal cortical GM, little consideration has been given to the degree of damage to and destruction of major connections between discretely affected regions and the rest of his brain.

**Table 1:** Estimates of the Damage to Gage's Brain.

Examiner	Destroyed	Damaged
Harlow, 1848	Left frontal	Not stated
Phelps, 1849	Not stated	Not stated
Bigelow, 1850	Central left frontal, front of left temporal	Left ventricle, medial right frontal
Harlow, 1868	Left frontal only	Left lateral ventricle, part of left temporal
Dupuy, 1873	“absolue” left frontal	Not stated
Dupuy, 1877	Broca's area, Sylvian artery, Island of Reil	Not stated
Ferrier, 1877	Left frontal only	Not stated
Ferrier, 1878	Left prefrontal	Tip of left temporal
Hammond, 1871	Left anterior only, 3^rd^ frontal convolution and Island of Reil escaped	Not stated
Cobb, 1940, 1943	Large part of left and some right prefrontal	Large parts of left and some right prefrontal
Tyler and Tyler, 1982	Left anterior frontal, tip of left temporal, anterior horn of left lateral ventricle, head of caudate nucleus and putamen, right hemisphere – including right superior and cingulated gyri	
H. Damasio et al., 1994	Anterior half of left orbital frontal cortex, polar and anterior mesial frontal cortices, anterior-most part of anterior cingulated gyrus, right hemisphere similar but less marked in orbital frontal region	Medial and superior right frontal
Ratiu and Talos 2004	Limited to the left frontal lobe and spared the superior sagittal sinus	

See MacMillan, *An Odd Kind of Fame*, for references to Tyler and Tyler.

WM fasciculi link activity between cortical areas of the brain [9], [10], become systematically myelinated through brain maturation [11], govern fundamental cognitive systems [12], and may be disrupted in neurological [13] and psychiatric disease [14]. Penetrative TBI in cases of wartime [15], industrial [16], gunshot [17], or domestic [18] injury often result in significant damage to brain connectivity, loss of function, and often death. Yet, in some instances, recovery from objects penetrating WM [19] have been reported with minimal sequelae [20]. Neuroimaging studies of WM tracts in TBI have revealed not only significant acute damage to fiber pathways but also that measures of fiber integrity can show partial fiber recovery over time [21], presumably due to cortical plasticity [22] in non-penetrative cases.

Given recent interest in the atlasing of the human WM connectome (e.g. http://www.humanconnectomeproject.org), a detailed consideration of the putative damage to Mr. Gage's connectomics and implications for changes in behavior is provocative and compelling. Nerve damage is superficially evident through reports of eventual loss of sight in Gage's left eye, left eyelid ptosis [23], and recognition of potential WM damage by other investigators [7]. Further examination of the extent of Gage's WM damage and of its effects on network topology and regional connectedness can offer additional context into putative behavioral changes. Due to the absence of original brain tissue and to the lack of a recorded autopsy from this case, one can only estimate the extent of damage from bony structures and can never be confident concerning which precise brain tissues were impacted. However, brain tissue *in situ* from a representative population can be considered and it can be assumed that Mr. Gage's anatomy would have been similar. In this examination, we obtained the original high-resolution CT data of the Gage skull used by Ratiu *et al.*, and computationally estimated the best-fit rod trajectory through the skull. Via multimodal analysis of T1-weighted anatomical MRI and DWI in N = 110 normal, right-handed males, aged 25–36, we quantify the extent of acute regional cortical loss and examine in detail the expected degree of damage to Mr. Gage's WM pathways.

## Results

**Figure 2:**
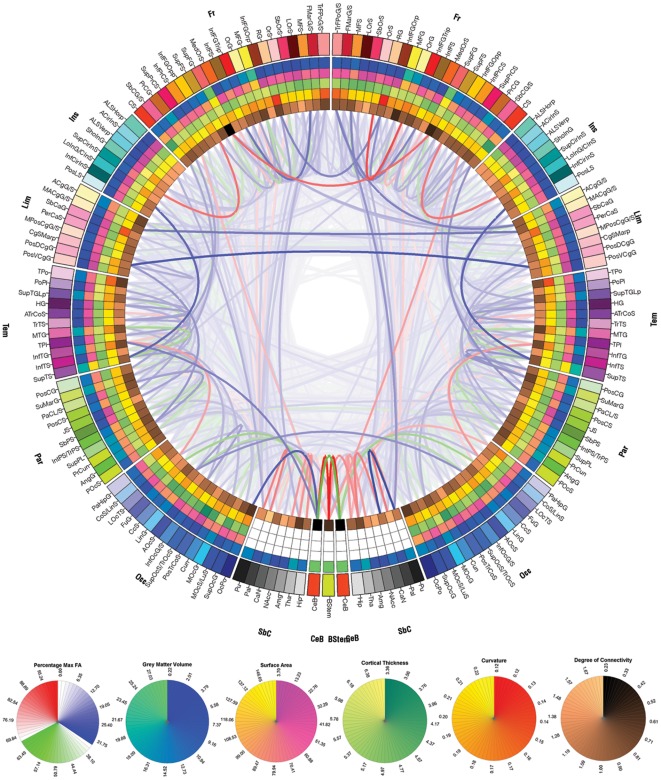
The circular representation of cortical anatomy and WM connectivity from N = 110 normal right-handed males (age 25–36). The outermost ring shows the various brain regions arranged by lobe (fr – frontal; ins – insula; lim – limbic; tem – temporal; par – parietal; occ- occipital; nc – non-cortical; bs – brain stem; CeB - cerebellum) and further ordered anterior-to-posterior based upon the centers-of-mass of these regions in the published Destrieux atlas [72] (see also Table 6 for complete region names, abbreviations, and FreeSurfer IDs, and Table 7 for the abbreviation construction scheme). The left half of the connectogram figure represents the left-hemisphere of the brain, whereas the right half represents the right hemisphere with the exception of the brain stem, which occurs at the bottom, 6 o'clock position of the graph. The lobar abbreviation scheme is given in the text. The color map of each region is lobe-specific and maps to the color of each regional parcellation as shown in Fig. S2. The set of five rings (from the outside inward) reflect average i) regional volume, ii) cortical thickness, iii) surface area, and iv) cortical curvature of each parcellated cortical region. For non-cortical regions, only average regional volume is shown. Finally, the inner-most ring displays the relative degree of connectivity of that region with respect to WM fibers found to emanate from this region, providing a measure of how connected that region is with all other regions in the parcellation scheme. The links represent the computed degrees of connectivity between segmented brain regions. Links shaded in blue represent DTI tractography pathways in the lower third of the distribution of fractional anisotropy, green lines the middle third, and red lines the top third. Circular “color bars” at the bottom of the figure describe the numeric scale for each regional geometric measurement and its associated color on that anatomical metric ring of the connectogram.

Computationally projecting a model of the tamping iron through the T1 MRI anatomical volumes warped to the Gage skull geometry (Table 2; Fig. 1b–c; see also Methods) in light of previously reported anatomical constraints (Table 3) and healthy brain morphometry and connectivity (Fig. 2), the average percentage of total cortical GM volume intersected was 3.97±0.29% (mean±SD), where the cortical regions most affected by the rod (>25% of their regional volumes) included (mean±SD): the left orbital sulcus (OrS; 90.86±6.97%), the left middle frontal sulcus (MFS; 80.33±10.01), the horizontal ramus of the anterior segment of the lateral sulcus (ALSHorp; 71.03±22.08%), the anterior segment of the circular sulcus of the insula (ACirInS; 61.81±18.14%), the orbital gyrus (OrG; 39.45±6.17%), the lateral orbital sulcus (LOrS; 37.96±20.24%), the superior frontal sulcus (SupFS; 36.29±12.16%), and the orbital part of the inferior frontal gyrus (InfFGOrp; 28.22±19.60%). While extensive damage occurred to left frontal, left temporal polar, and insular cortex, the best fit rod trajectory did not result in the iron crossing the midline as has been suggested by some authors (see Methods). As a result, no direct damage appeared to occur in right frontal cortices as evident from our representative sample cohort. A complete list of all cortical areas experiencing damage is listed in Table 4.

**Table 2:** Gage Skull Measurements.

	Exterior^1^	Interior^2^	Foramen Magnum^3^	Zygomatic Arch^4^
**Skull**	189 mm	100 mm	30 mm	125.9 mm
Injury Measures	Bottom	Clockwise from	Outside	
External Edges of Anterior Wound Bone	32.5 mm	42.7 mm	42.8 mm	36.2 mm
External Edges of Anterior Wound Hole	35 mm	63.3 mm	45.6 mm	39.2 mm
**Distances**	Inferior Left	Clockwise from	Outside	
From Anterior Wound Corners to Nasion	60 mm	102 mm	83 mm	58.4 mm
From Most Superior Portion of Anterior Crack to Anterior Wound Corners	31.5 mm	58 mm	42 mm	2 mm
From Most Anterior Portion of Superior Crack to Anterior Wound Corners	60 mm	0 mm	42 mm	58 mm
	(External) Left Inferior to Right Superior	(External) Right Inferior to Medial Superior		
Diagonal Length of Anterior Wound	65.3 mm	47.2 mm		

1 Maximum length of the skull.

2 Maximum height of the skull above the inion-glabella line.

3 Inion-glabella line to floor of the middle fossa at the edge of the Foramen Magnum.

4 Maximum width of the skull at the level of the zygomatic arches.

**Table 3:** Descriptions of Damage to Gages' Skull Serving as Constraints for Determining the Trajectory of the Tamping Iron.

Harlow	Bigelow: Correspondence of Dr. Williams	Bigelow: During Gage's Life	Damasio	Ratiu and Talos
**Protruding the globe of the left eye from its socket, by nearly one half its diameter.**	Inverted Funnel 2 inches in every direction	Linear Cicatrix of an inch in length occupies the left ramus of the jaw near its angle	Mandible intact	the optic canal was spared and the eyeball and the left optic nerve stayed medially
**it penetrated the integuments, the masseter and temporal muscles, passed under the zygomatic arch, and (probably) fracturing the temporal portion of the sphenoid bone, and the floor of the orbit of the left eye, entered the cranium, passing through the anterior left lobe of the cerebrum, and made its exit in the median line, at the junction of the coronal and sagittal sutures, lacerating the longitudinal sinus, fracturing the parietal and frontal bones extensively,**	Size of the hole about that of the rod, although this may take the funnel (hinging) into account	thickening of tissue about the malar bone	Zygomatic arch was mostly intact but had a chipped area - medial and superior edge (grazed)	as the iron's tapered end penetrated the left cheek, it fractured the maxilla and the sphenoid wings. As it passed through the orbit, the left half of the bony face swung laterally
**A portion of the anterior superior angle of each parietal bone, and a semi-circular piece of the frontal bone, were fractured, leaving a circular opening of about 3+1/2 inches in diameter**	Initially missed the facial wound, had to be pointed out by Gage	left eye considerably more prominent, incapable of outward/upward motion, but other motions unimpaired	Last superior molar socket intact, but tooth missing	Anterior to the cingulate gyrus and to the left ventricle.
**Lost vision 10 days after accident**	Slit from the angle of the jaw forward 1.5 inch, very stretched laterally, and appeared discolored by powder and rust	Irregular and deep sulcus several inches in length, beneath which the pulsations of the brain can be perceived	No closer than 1.5 cm from the mid thickness of the zygomatic arch	SSS not ruptured: “No rhinoliquorhea or other indication for post-traumatic CSF fistula was reported”
			1 cm from the last superior molar	Point out crack in zygomatic bone, however this looks to be in the same spot as the natural suture between the zygomatic and the maxilla
			0.5 cm from the coronoid process of the mandible	actual bone loss at the iron's point of entry into the skull as well as in the iron's path through the orbit and the sphenoid is approximately 50% smaller than the max- imum diameter of the iron. Since the edges of the region of bone loss show little evidence of healing—mostly a few small osteophytes with no considerable callus for- mation, it follows that portions of the skull lateral to the iron must have fractured an hinged open as the iron passed through, and were then drawn back into place elas- tically and spontaneously realigned by the soft tissue
			Could not have hit anterior horn of lateral ventricle	healed fracture line that runs downward from the inferior orbital rim through the inferior orbital foramen, to the alveolar crest above the second molar (fr)
				because the trajectory of the iron went from the left cheek to the midline of the frontal bone above the orbit, the iron must have passed solely through the fronto-orbital and prefrontal cortex in the left hemisphere

**Table 4:** Percentage of each cortical region impacted by the passage of the rod.

*Regional Parcellation Coding*	*Mean Percentage of Regional Loss*	*Standard Deviation*
*OrS*	90.8642	6.9738
MFS	80.3275	10.0096
ALSHorp	71.0288	22.0750
ACirIns	61.8052	18.1382
OrG	39.4491	6.1727
LOrs	37.9640	20.2393
SupFS	36.2909	12.1636
InfFGOrp	28.2180	19.6026
InfES	24.3090	10.3200
ACgG/S	23.2818	8.5984
MFG	21.0579	5.5363
SupFG	16.7034	4.2277
TPro	15.9092	13.7996
SupCirIns	13.1850	5.1297
FMarG/S	10.8534	8.1461
MedOrS	7.8242	8.09648
ShoInG	6.5450	6.2824
ALSVerp	5.7157	10.6281
PoPl	5.4607	8.6884
SbOrs	3.6767	6.1917
InfFGTrip	3.4548	4.3119
SupTGLp	2.5458	3.8131
TrFPoG/S	1.7822	6.0115
Left-Can	0.9526	1.2567
MACgG/S	0.8396	2.2467
RG	0.5484	2.0909
PerCaS	0.1570	0.8941
InfFGOpp	0.1303	0.5813
MTG	0.0932	0.5224
Left-Pu	0.0513	0.2833
InfCirInS	0.0316	0.2170
InfTG	0.0150	0.1492
SupFG(rh)	0.0043	0.0285
InfTS	0.0034	0.0365
SupTS	0.0007	0.0076
ACgG/S(rh)	0.0002	0.0023
**Total White Matter**	**10.7155**	**5.4566**
**Total Grey Matter**	**3.9718**	**0.2995**

**Figure 3:**
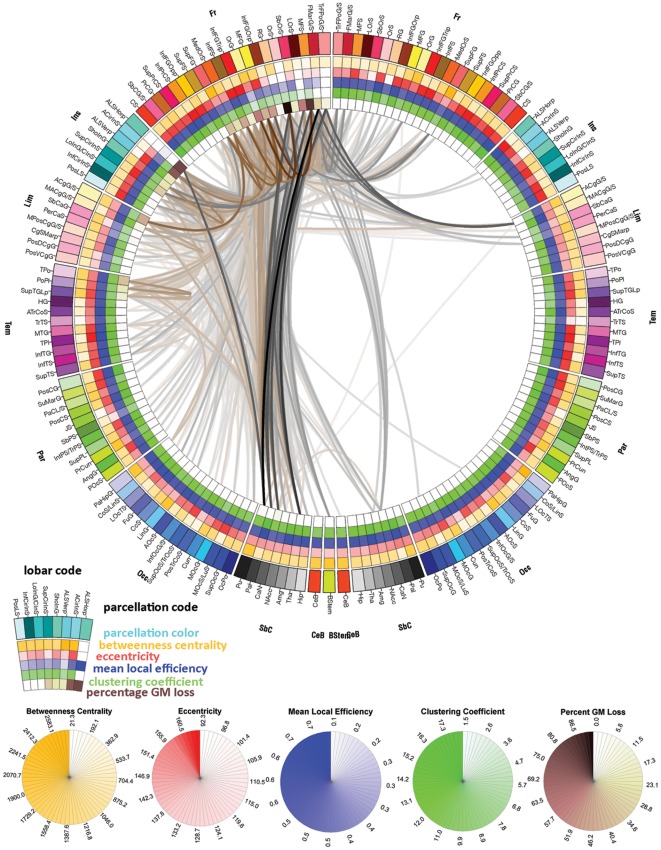
Mean connectivity affected by the presence of the tamping iron combined across subjects. The lines in this connectogram graphic represent the connections between brain regions that were lost or damaged by the passage of the tamping iron. Fiber pathway damage extended beyond the left frontal cortex to regions of the left temporal, partial, and occipital cortices as well as to basal ganglia, brain stem, and cerebellum. Inter-hemispheric connections of the frontal and limbic lobes as well as basal ganglia were also affected. Connections in grayscale indicate those pathways that were completely lost in the presence of the tamping iron, while those in shades of tan indicate those partially severed. Pathway transparency indicates the relative density of the affected pathway. In contrast to the morphometric measurements depicted in Fig. 2, the inner four rings of the connectogram here indicate (from the outside inward) the regional network metrics of betweenness centrality, regional eccentricity, local efficiency, clustering coefficient, and the percent of GM loss, respectively, in the presence of the tamping iron, in each instance averaged over the N = 110 subjects.

**Figure 4:**
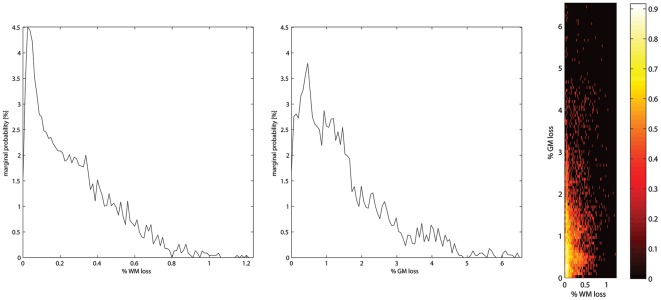
The distribution characteristics of affected white matter pathways. WM fiber pathways intersected by the rod were pooled across all N = 110 subjects and examined for a) the relative lengths (w_ij_) of affected pathways and b) the relative percentages of lost fiber density (g_ij_); c) the bivariate distribution of g_ij_ versus w_ij_ indicating that local fiber pathways were affected, *e.g.* relatively short pathways proximal to the injury site, as well as damaging dense, longer-range fiber pathways, *e.g.* innervating regions some distance from the tamping iron injury (see “*Calculation of Pathology Effects upon GM/WM Volumetrics*” for further details).

The amount of total WM volume lost due to the tamping iron was 10.72±5.46% (mean±SD). Examination of lesioned connectivity matrices indicated that fiber bundles from nearly the entire extent of the left frontal cortex were impacted by the presence of the tamping iron (e.g. Fig. 1d), which in turn affected most of that hemisphere as well as contralateral regions (Fig. 3). The effect of this lesion on network properties was assessed 1) with respect to the healthy intact network, generally, as well as 2) in contrast to the average effects of similarly-sized lesions simulated elsewhere in the cortex, as related to local GM loss as well as distributed loss of connectivity (Fig. 4a–c). Metrics representative of three specific global network attributes were examined: characteristic path length (λ, measuring network integration), mean local efficiency (e, segregation), and small worldness (S) (Table 5).

**Table 5:** Comparison of Intact, Tamping Iron, and Simulated Network Attributes.

Network Type	Integration (Characteristic Path Length, *λ*)	Segregation (Mean Local Efficiency, *e*)	Small Worldness (*S*)
**Intact (I)**	*λ_Obs_(I)* = 7.7222±3.0227 *λ_Rand_(I)* = 5.6377±2.1899 *λ_Obs_(I)/λ_Rand_(I)* = 1.3697±0.0534	*e_Obs_(I)* = 0.4824±0.1077 *e_Rand_(I)* = 0.0805±0.0443*e_Obs_(I)/e_Rand_(I)* = 6.8953±2.1672	*S* = 3.7226±1.0778
**Tamping Iron (T)** 1	*λ_Obs_(T)* = 7.8893±3.0817 *λ_Obs_(T)/λ_Rand_(I)* = 1.3987±0.0532^b^	*e_Obs_(T)* = 0.4607±0.1104 *e_Obs_(T)/e_Rand_(I)* = 5.7229±2.0538^c^	*S* = 3.7289±0.9853^a^
**Simulated Lesions (L)** 2	*λ_Obs_(L)* = 8.3826±3.3196 *λ_Obs_(L)/λ_Rand_(I)* = 1.4869±0.0469^d^	*e_Obs_(L)* = 0.4352±0.1054 *e_Obs_(L)/e_Rand_(I)* = 5.4062±1.5321^d^	*S* = 3.6061±0.7094^c^

a T vs. I: *p(t)* = ns.

b T vs. L: *p(t)*≤0.0001.

c T vs. I: *p(t)*≤0.001.

d L vs. I: *p(t)*≤0.0001.

1 Means and standard deviations are reported as computed over N = 110 subjects included in the study (see text for details). Paired-sample Student's t-tests were used to compare the damaged and intact networks; subscripts refer to “observed” (Obs) and “random” (Rand); df = 109.

2 Means and standard deviations are reported as computed over N = 110 subjects included in the study, after first averaging metric values over 500 simulated lesions of the cortex (see text for details).

**Figure 5:**
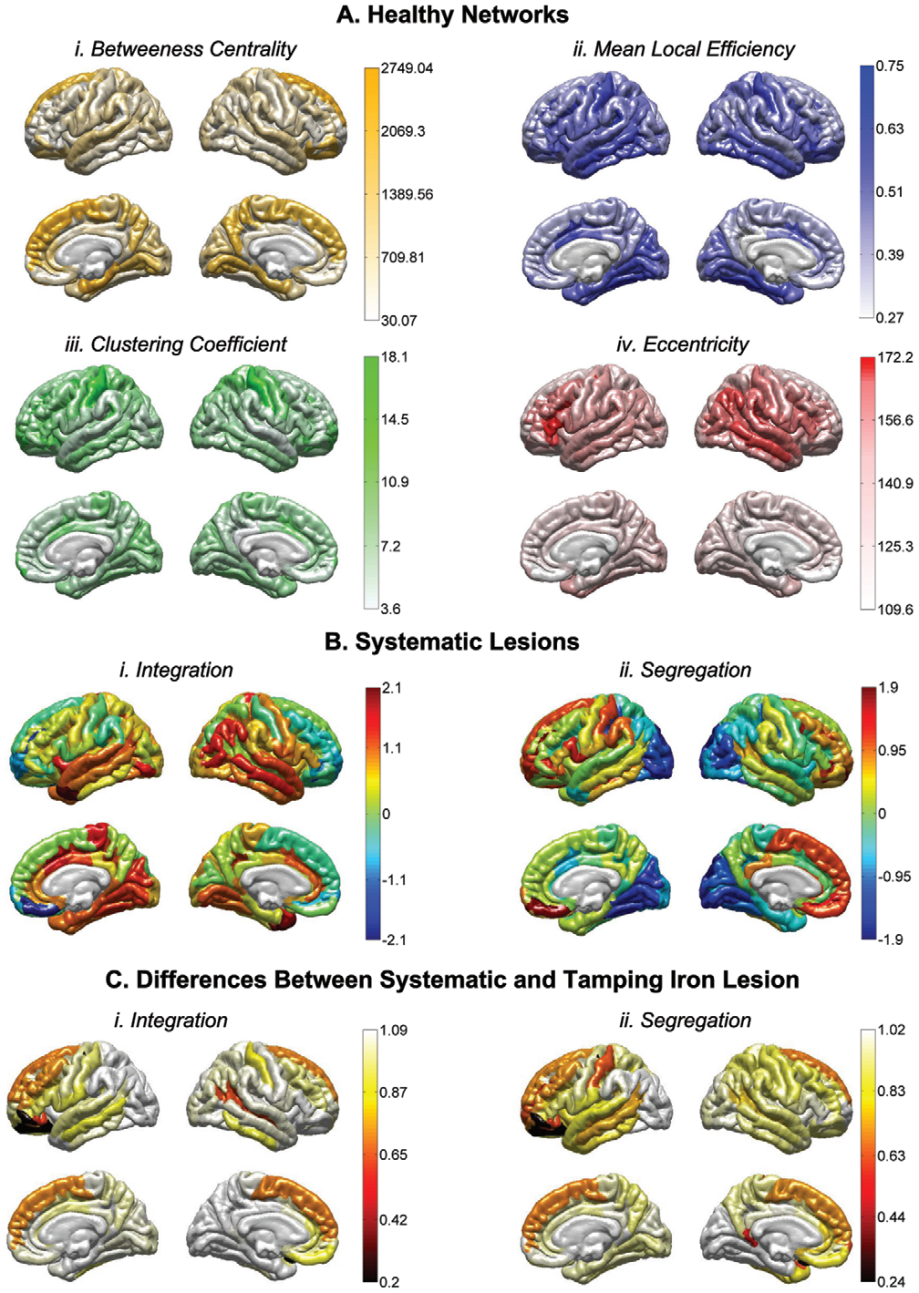
Healthy region-specific graph theoretical metrics, the effects of systematic lesions, and the difference between the observed and simulated tamping iron lesions. A) Cortical maps of regional graph theoretical properties. Regions affected by the passage of the tamping iron include those having relatively high betweenness centrality and clustering coefficients but relatively low mean local efficiency and eccentricity. B) A cortical surface schematic of the relative effects of systematic lesions of similar WM/GM attributes over the cortex for both network integration (i) and segregation (ii). For each mapping, colors represent the Z-score difference between systematic lesions of that area relative the average change in integration taken across all simulated lesions. C) Cortical maps of the differences/similarity between the effects on integration and segregation observed from the tamping iron lesion with that of each simulated lesion. Here black is most similar (e.g. the observed lesion is most similar to itself) whereas white is least similar to (e.g. most different from) the tamping iron's effects on these measures of network architecture.

Tables 6 and 7 provide details on the regional coding used for brain parcellation which were subjected to estimation of the effects of the tamping iron, lesion simulation modeling, and which encode the text on the outer-most rings of Figs. 2 and 3. Differences in measures of network connectivity due to the rod's passage were apparent in terms of network integration, segregation, but not small worldness as compared to the unlesioned, healthy network. Specifically, when removing those cortical areas and fiber pathways intersected by the iron, characteristic path length was found to be significantly decreased in Gage compared to the intact network (*p*≤0.0001), mean local efficiency was decreased (*p*≤0.0001), while small worldness showed no statistical difference (*p*≤0.9467, ns). Regionally-specific network theoretical metrics in the affected regions and those to which they connect were also affected (see Fig. 5A). This suggests that, not surprisingly, with significant loss of WM connectivity between left frontal regions and the rest of the brain, the surviving network of brain was likely to have been heavily impaired and its functions considerably compromised.

**Table 6:** Regional Parcellation Coding Scheme.

*Abbreviation*	*Description*	*FreeSurfer Code*	*RGB Code*
NEOCORTICAL STRUCTURES			
ACgG/S	anterior part of the cingulate gyrus and sulcus	G_and_S_cingul-Ant	255 255 180
ACirInS	anterior segment of the circular sulcus of the insula	S_circular_insula_ant	102 255 255
ALSHorp	horizontal ramus of the anterior segment of the lateral sulcus (or fissure)	Lat_Fis-ant-Horizont	0 255 204
ALSVerp	vertical ramus of the anterior segment of the lateral sulcus (or fissure)	Lat_Fis-ant-Vertical	0 255 255
AngG	angular gyrus	G_pariet_inf-Angular	0 255 0
AOcS	anterior occipital sulcus and preoccipital notch (temporo-occipital incisure)	S_occipital_ant	51 51 255
ATrCoS	anterior transverse collateral sulcus	S_collat_transv_ant	153 0 204
CcS	calcarine sulcus	S_calcarine	102 153 255
CgSMarp	marginal branch (or part) of the cingulate sulcus	S_cingul-Marginalis	255 192 201
CoS/LinS	medial occipito-temporal sulcus (collateral sulcus) and lingual sulcus	S_oc-temp_med_and_Lingual	153 204 255
CS	central sulcus (Rolando's fissure)	S_central	255 51 0
Cun	cuneus (O6)	G_cuneus	0 153 255
FMarG/S	fronto-marginal gyrus (of Wernicke) and sulcus	G_and_S_frontomargin	204 0 51
FuG	lateral occipito-temporal gyrus (fusiform gyrus, O4-T4)	G_oc-temp_lat-fusifor	102 102 255
HG	Heschl's gyrus (anterior transverse temporal gyrus)	G_temp_sup-G_T_transv	102 0 102
InfCirInS	inferior segment of the circular sulcus of the insula	S_circular_insula_inf	0 102 102
InfFGOpp	opercular part of the inferior frontal gyrus	G_front_inf_Opercular	255 204 0
InfFGOrp	orbital part of the inferior frontal gyrus	G_front_inf-Orbital	153 051 0
InfFGTrip	triangular part of the inferior frontal gyrus	G_front_inf-Triangul	255 0 0
InfFS	inferior frontal sulcus	S_front_inf	153 102 0
InfOcG/S	inferior occipital gyrus (O3) and sulcus	G_and_S_occipital_inf	51 153 255
InfPrCS	inferior part of the precentral sulcus	S_precentral-inf-part	255 153 0
IntPS/TrPS	intraparietal sulcus (interparietal sulcus) and transverse parietal sulci	S_intrapariet_and_P_trans	51 255 51
InfTG	inferior temporal gyrus (T3)	G_temporal_inf	255 0 255
InfTS	inferior temporal sulcus	S_temporal_inf	204 0 153
JS	sulcus intermedius primus (of Jensen)	S_interm_prim-Jensen	153 204 0
LinG	lingual gyrus, lingual part of the medial occipito-temporal gyrus (O5)	G_oc-temp_med-Lingual	102 204 255
LOcTS	lateral occipito-temporal sulcus	S_oc-temp_lat	153 153 255
LoInG/CInS	long insular gyrus and central insular sulcus	G_Ins_lg_and_S_cent_ins	0 204 204
LOrS	lateral orbital sulcus	S_orbital_lateral	102 0 0
MACgG/S	middle-anterior part of the cingulate gyrus and sulcus	G_and_S_cingul-Mid-Ant	255 240 191
MedOrS	medial orbital sulcus (olfactory sulcus)	S_orbital_med-olfact	255 102 0
MFG	middle frontal gyrus (F2)	G_front_middle	255 255 051
MFS	middle frontal sulcus	S_front_middle	255 153 51
MOcG	middle occipital gyrus (O2, lateral occipital gyrus)	G_occipital_middle	0 204 244
MOcS/LuS	middle occipital sulcus and lunatus sulcus	S_oc_middle_and_Lunatus	0 51 255
MPosCgG/S	middle-posterior part of the cingulate gyrus and sulcus	G_and_S_cingul-Mid-Post	255 224 203
MTG	middle temporal gyrus (T2)	G_temporal_middle	255 102 204
OcPo	occipital pole	Pole_occipital	0 0 153
OrG	orbital gyri	G_orbital	255 255 153
OrS	orbital sulci (H-shaped sulci)	S_orbital-H_Shaped	255 204 204
PaCL/S	paracentral lobule and sulcus	G_and_S_paracentral	204 255 153
PaHipG	parahippocampal gyrus, parahippocampal part of the medial occipito-temporal gyrus (T5)	G_oc-temp_med-Parahip	204 204 255
PerCaS	pericallosal sulcus (S of corpus callosum)	S_pericallosal	255 164 200
POcS	parieto-occipital sulcus (or fissure)	S_parieto_occipital	204 255 51
PoPl	polar plane of the superior temporal gyrus	G_temp_sup-Plan_polar	204 153 255
PosCG	postcentral gyrus	G_postcentral	204 255 204
PosCS	postcentral sulcus	S_postcentral	153 255 0
PosDCgG	posterior-dorsal part of the cingulate gyrus	G_cingul-Post-dorsal	255 175 201
PosLS	posterior ramus (or segment) of the lateral sulcus (or fissure)	Lat_Fis-post	204 255 255
PosTrCoS	posterior transverse collateral sulcus	S_collat_transv_post	51 102 255
PosVCgG	posterior-ventral part of the cingulate gyrus (isthmus of the cingulate gyrus)	G_cingul-Post-ventral	255 208 202
PrCG	precentral gyrus	G_precentral	204 102 0
PrCun	precuneus (medial part of P1)	G_precuneus	204 255 0
RG	straight gyrus (gyrus rectus)	G_rectus	255 204 153
SbCaG	subcallosal area, subcallosal gyrus	G_subcallosal	255 153 200
SbCG/S	subcentral gyrus (central operculum) and sulci	G_and_S_subcentral	255 102 153
SbOrS	suborbital sulcus (sulcus rostrales, supraorbital sulcus)	S_suborbital	255 51 102
SbPS	subparietal sulcus	S_subparietal	102 153 0
ShoInG	short insular gyri	G_insular_short	51 255 204
SuMarG	supramarginal gyrus	G_pariet_inf-Supramar	204 255 102
SupCirInS	superior segment of the circular sulcus of the insula	S_circular_insula_sup	0 153 153
SupFG	superior frontal gyrus (F1)	G_front_sup	255 102 102
SupFS	superior frontal sulcus	S_front_sup	204 153 0
SupOcG	superior occipital gyrus (O1)	G_occipital_sup	0 0 255
SupPrCS	superior part of the precentral sulcus	S_precentral-sup-part	255 0 102
SupOcS/TrOcS	superior occipital sulcus and transverse occipital sulcus	S_oc_sup_and_transversal	0 102 255
SupPL	superior parietal lobule (lateral part of P1)	G_parietal_sup	153 255 153
SupTGLp	lateral aspect of the superior temporal gyrus	G_temp_sup-Lateral	153 51 255
SupTS	superior temporal sulcus	S_temporal_sup	204 51 255
TPl	temporal plane of the superior temporal gyrus	G_temp_sup-Plan_tempo	153 0 153
TPo	temporal pole	Pole_temporal	255 204 255
TrFPoG/S	transverse frontopolar gyri and sulci	G_and_S_transv_frontopol	255 153 153
TrTS	transverse temporal sulcus	S_temporal_transverse	255 153 255
SUB-CORTICAL STRUCTURES			
Amg	Amygdala	Amygdala	159 159 159
CaN	caudate nucleus	Caudate	96 96 96
Hip	hippocampus	Hippocampus	223 223 223
NAcc	nucleus accumbens	Accumbens-area	128 128 128
Pal	Pallidum	Pallidum	64 64 64
Pu	Putamen	Putamen	32 32 32
Tha	Thalamus	Thalamus-Proper	191 191 191
CEREBELLUM AND BRAIN STEM			
CeB	cerebellum	Cerebellum-Cortex	255 64 0
BStem	brain stem	Brain-Stem	207 255 48

**Table 7:** Regional Parcellation Coding Scheme.

Abbreviation	Keyword	Abbreviation	Keyword
A	Anterior	Med	Medial
Acc	Accumbens	Mar	Marginal
Ang	Angular	N	Nucleus
B	Brain	Oc	Occipital/occipito-
C	Central	Op	Opercular
Ca	Callosal	Or	Orbital
Cau	Caudate	P	Parietal
Cc	Calcarine	Pa	Para-
Ceb	Cerebellum	Pal	Palladium
Cg	Cingulate	Per	Peri-
Cir	Circular	Pl	Plane
Cla	Claustrum	Po	Pole/polar
Co	Collateral	Pos	Posterior/post-
Cun	Cuneus	Pr	Pre-
D	Dorsal	Pu	Putamen
F	Frontal/fronto-	p	Part
Fu	Fusiform	pl	Plane
G	Gyrus/gyri	R	Rectus
H	Heschl	S	Sulcus/sulci
Hip	Hippocampus/hippocampal	Sb	Sub-
Hor	Horizontal	Sho	Short
In	Insula/insular	Su	Supra-
Inf	Inferior	Sup	Superior
Int	Intra-	T	Temporal
J	Jensen	Tha	Thalamus
L	Lateral/lobule	Tr	Transverse
Lin	Lingual	Tri	Triangular
Lu	Lunate/lunatus	V	Ventral
Lo	Long	ver	Vertical
M	Middle		

To further provide a baseline for comparison of the tamping iron lesion against similarly-sized lesions located elsewhere in the cortex, we conducted a systematic random simulation of 500 similarly-sized lesions across our N = 110 healthy subject cohort. The network containing the lesion due to the tamping iron was systematically compared against the distributions of the above mentioned metrics from the simulated lesion set. When paired *t*-statistics were computed to determine whether tamping iron lesion differed significantly from the standpoint of network metric values, standardized with respect to the intact network, as compared to other brain lesions of the same size, the characteristic path length (integration), mean local efficiency (segregation), and small worldness, while significantly different from that of the intact networks, were not found to be more severe than the average network properties of average similarly sized GM/WM lesion. These results are summarized in Fig. 5B and 5C. These indicate that alterations to network integration resulting from the tamping iron lesion resulted in greater average path length than that of the intact network but which was less than the average effects of other equally sized lesions. Likewise, segregation, as measured using mean local efficiency, was reduced compared to the intact network, but greater than the average effects of the simulated lesions. These results suggest that Mr. Gage's lesion, while severe and certain to have affected WM connectivity in his left cerebral hemisphere and throughout his brain, could have been considerably more severe had the tamping iron pierced other areas of his brain.

## Discussion

The case of Phineas Gage is among the most famous and infamous in the history of brain science. The interpretations of his incredible injury and attempts to characterize it have been ongoing since soon after it occurred. Our consideration sought to provide a modern connectomic understanding of Mr. Gage's injury and put it into context as involving brain WM in addition to the GM damage discussed by other authors. While we, too, are constrained by the relics left from Mr. Gage's life and what evidence can be gleaned from them, work detailed in this article differs considerably from previous examinations of this case and topic in several key areas: we 1) precisely model the trajectory of the tamping iron through high resolution computed tomographic data of Mr. Gage's skull - a rare imaging data set that, until now, had been lost to science for over a decade; 2) geometrically fit N = 110 age, gender, and handedness matched modern subject MRI brain volumes into the Gage cranial vault to assess average cortical metrics and their degree of variability; 3) in so doing, illustrate that while ∼4% of the cortex was intersected by the rod's passage, ∼11% of total white matter was also damaged, and provide estimates of the degree of damage experienced under a well-established brain parcellation scheme; 4) map high angular resolution diffusion neuroimaging tractography into the same space to measure damage to the pair-wise connections between atlas-defined cortical regions; and 5) compare the graph theoretical properties of the observed lesion against those expected from theoretically similar lesions systematically located throughout the brain. In what follows, we comment on our approach and findings.

### Trajectory of the Tamping Iron

Various descriptions of the trajectory of the tamping iron through the Mr. Gage's skull have been given, which has understandably led to differing opinions about which parts of his brain were subjected to damage. Harlow, the physician responsible for Gage's initial treatment, documented that only the left hemisphere had been affected while the right remained unaffected [24]. In contrast, Bigelow maintained that some right-sided damage must have occurred. Dupuy [25] agreed with the left sidedness of the trajectory but placed it more posterior, claiming that motor and language areas had been destroyed – supporting the anti-localizationist arguments popular of the era. Ferrier [26], illustrating that the motor and language areas had been spared, concluded that damage was limited only to the left hemisphere – a conclusion later echoed by Cobb [27]. In their measurements, Damasio et al. estimated the damage to be more frontal and right sided, whereas Ratiu and colleagues concluded that damage was limited to the left frontal lobe and that it did not cross the midline. Central to these differences in interpretation is likely to be how mandible position has been considered. To satisfy the observed anatomical constraints with the mouth closed would result in a greater right-sided inclination of the rod. Yet, as Harlow originally noted, Gage was in the act of speaking to his men at the moment of the injury and, thus, his mouth was likely open. We observe that with the jaw opened, the best-fit rod trajectory satisfying all constraints does not intersect or cross the superior sagittal sulcus and the injury is specific to the left frontal lobe. Thus, our conclusions are congruent with those of Harlow, Ferrier, as well as with those of Ratiu and Talos and, given the detailed computational approach taken, seem to provide the most likely reconstruction of the acute damage caused by the tamping iron.

### Alterations of Network Connectivity Due to the Tamping Iron

The loss of ∼11% total WM volume in the left frontal lobe suggests that the iron's effects on Mr. Gage's brain extended well beyond the loss of left frontal GM alone. Overall differences in metrics of network integration as well as segregation were observed relative to intact connectivity, suggesting widespread disruption of networks involving damage to the left frontal and temporal pathways. Alterations indicate major changes to global network topology which affected network-wide efficiency. In the healthy cohort examined here, the region-to-region WM connectedness when in the presence of the rod was found to be associated with several important fiber bundles. Specifically, connectivity was affected between the frontal lobes and the basal ganglia, the insula, limbic, and other major lobes of the left hemisphere, in addition to right frontal, insular, and limbic areas. This severed portions of the uncinate fasciculus (UF) - connecting parts of the limbic system such as the hippocampus and amygdala in the temporal lobe with frontal regions such as the orbito-frontal cortex. The cingulum bundle - the collection of WM fibers projecting from the cingulate gyrus to the entorhinal cortex, allowing for communication between components of the limbic system – was also damaged. Additionally, the superior longitudinal fasciculus (SLF) was impacted – the long bi-directional bundles of neurons connecting the rostral and caudal aspects of the cerebrum in which each association fiber bundle is lateral to the centrum ovale linking the frontal, occipital, parietal, and temporal lobes. Fibers here pass from the frontal lobe through the operculum to the posterior end of the lateral sulcus, where numerous processes radiate into the occipital lobe while others turn downward and forward around the putamen and project to anterior portions of the temporal lobe. The occipito-temporal projection [28] in humans connects the temporal lobe and occipital lobe, running along the lateral walls of the inferior and posterior cornua of the lateral ventricle. The connectivity of the orbital cortex with temporal lobe regions via the UF which is among the last to complete myelination in development [29], has been shown to be particularly affected in patients with mental illness [30], and to be related to cognitive deficits in TBI [31], [32]. WM fascicular damage in these instances was likely an important factor in Gage's reported post-injury symptomatology as well as in his reported and putative behavioral issues.

The obtained results suggest that GM damage had wider reaching influence than previously described and compromised several aspects of Gage's network of WM connectivity. Regions whose connectivity within and between cerebral hemispheres were affected included: the left frontal lobe (the transverse fronto-polar gyrus, fronto-marginal gyrus, middle frontal gyrus, lateral orbital sulcus, orbital sulcus, oribital part of the inferior frontal gyrus, triangular part of the inferior frontal gyrus, inferior frontal sulcus, medial orbital sulcus, orbital gyri, superior frontal gyrus, and opercular part of the inferior frontal gyrus); left insular cortex (horizontal and vertical ramus of the anterior segment of the lateral sulcus/fissure, the anterior/inferior/superior segments of the circular sulcus of the insula, short insular gyri, and long insular gyrus and central insular sulcus); and the left temporal lobe (the temporal pole and polar plane of the superior temporal gyrus) (Fig. 3). The marginal and bivariate probability distributions of average brain-normalized WM fiber bundle length (w_ij_) and proportion of GM density lost (g_ij_; Fig. 4a–c) unsurprisingly indicated a considerable number of relatively short connections being affected locally by the presence of the rod while, additionally, a considerable number of longer fiber bundles connecting relatively large regions of cortex were also impacted.

Alterations to these connections contribute to the significant reductions in characteristic path length and mean local efficiency of the remaining network after removal of the affected fibers. That no significant difference was observed concerning the small worldness of the tamping iron network as compared to the intact network suggest that a lesion of this size and scope, while severe, may not had appreciable effects on the degree of clustering of unaffected nodes Mr. Gage's brain relative to randomly degree-equivalent versions of that network. On the other hand, the average simulated lesion did show a significant reduction in small worldness, indicating that regions other than those affected may have more influence over the degree of measured network clustering. Thus, Mr. Gage's unaffected network may have still maintained its small world architecture of nodal clustering and presumed functional integrity, despite loss of major frontal and temporal lobe participation in the system resulting in deficits to measures network integration and segregation.

Several previous articles have precisely investigated the direct effects of node deletions of various size on network connectivity and architecture [33], [34], [35]. In particular, the paper by Alstott *et al.* provides a detailed examination of simulated lesion effects on brain networks both in terms of lesion location and extent. In their study of structural and functional connectivity data from N = 5 healthy subjects, they found that lesions to midline areas resulted in more profound effects on various network metrics than do more lateral brain regions. As might be expected, the magnitude of change was dependent upon the number of nodes removed from the network and the manner in which they were removed. Their observations indicate that networks may be insensitive to lesions involving random node removal or where node removal was based only upon a node's degree of connectedness. However, network lesioning based upon the targeted removal of nodes having high betweenness centrality - a measurement of the number of shortest paths from all vertices to all others which pass through a given node - resulted in greater network vulnerability as evident from significant reductions in global efficiency in contrast to random lesioning. This result is particularly compelling in regards to assessing the robustness of cortical architecture in the face of brain damage to major network hubs localized proximal to the cortical midline.

There is little doubt that a tamping iron injury to central nodes of the frontal lobe would have severely impacted Gage's brain connectivity. Fig. 3 shows the extent of white matter damage and the effects on several measures of network connectivity, including regional betweenness centrality, local efficiency, clustering coefficient, and eccentricity. Note that this illustration differs from Fig. 2 in that the inner-most rings are now colored according to the respective average nodal connectivity metrics in the presence of cortical loss incurred from the tamping iron. Additionally, Fig. 5A illustrates the spatial distribution of these regional connectivity metrics over the cortex when pooled across the intact healthy networks from our sample (sub-cortex not shown). We note that, as observed by Alstott et al., areas of relatively high betweenness centrality tended to be located along the frontal midline. Other metrics show similar regional concentrations (Fig. 5Aii–iv). However, while intact frontal areas of both hemispheres show high betweenness centrality (Fig. 5Ai), the regions of tamping iron damage encompassed many other regions as well having relatively less betweenness centrality, *e.g.* TrFPoG/S, RG, SbCaG, TPo. Removal of these areas, as illustrated by the various metric rings in the left frontal segment of the connectogram in Fig. 3, has wide ranging effects on the regionally-specific network metrics in unaffected brain regions.

It is evident that removal of these areas produce significant effects on global metrics of network segregation and integration. However, from systematic lesion simulation using a similar extent of GM/WM involvement, the effects on Mr. Gage's network integration and segregation were not found to be more severe that that observed from the “average” lesion. Clearly, a larger lesion would have affected a greater number of network nodes including various hubs resulting in further deleterious effects on network integration and segregation. Moreover, a different lesion altogether would have possibly resulted in more outwardly obvious sensorimotor deficits. Located in occipital cortex, for instance, the lesion might have resulted in sensory-specific changes in connectivity (e.g. blindness), or one involving more of the sub-cortex and brain stem could have been more clinically serious and resulted in death. Nevertheless, the observed damage illustrates that severe network insult affecting the majority of left hemisphere connectivity as well as right hemispheric inter-connections, was experienced. Such damage can be expected to have had its influence over the normal functioning of many regions non-local to the injury and their subsequent connectivity as well.

Therefore, in light of these observations, it would be safe to conclude that 1) Mr. Gage's injury very likely destroyed portions of the central hub structure in left frontal midline structures as well as temporal pole and limbic structures which have extensive connectivity throughout the left hemisphere as well as inter-hemispherically, 2) that the tamping iron's passage did not specifically remove only the most central network hubs but a host of regions having a range of network properties, and 3) that such damage to important network hubs connection to other brain regions having secondary levels of centrality, clustering, etc. are likely to have combined to give rise to the behavioral and cognitive symptomatology originally reported by Harlow. Knowledge of Gage's affected connectivity help provide clarity and context for symptomatologies subsequently only inferred by others.

### Implications for Gage's Reported Behavioral Changes

Traumatic brain injury of the frontal cortices is often associated with profound behavioral alterations, changes mood [36], working memory [37] and planning deficits [38], [39], social functioning [40], among other cognitive symptoms [41], [42], [43], [44]. Alterations to functional connectivity have also been reported [45], [46] which, in addition to cortical damage, likely related to accompanying diffuse axonal injury [47], [48]. It is also worth noting neurodegenerative diseases, such as the leukodystrophies [49], Alzheimer's Disease (AD) [50], [51], and early-stage frontotemporal dementia (FTD) [52], also have effects on brain networks involving connectivity of the frontal lobe. Altered structural connectivity in these disorders illustrates changes in large-scale brain network organization deviating from healthy network organization [53], with possible effects on resting state connectivity [54]. Disruptions of WM connectivity are also known to underlie elements of psychiatric illness [55], [56], [57] which are associated with behavioral alterations not dissimilar to those reported in Mr. Gage.

In particular, network damage, predominantly of the left basal forebrain and of its connections throughout the left as well as into right frontal cortices, was particularly extensive. Processing of emotion stimuli have been associated with connectivity of the frontal cortex and amygdala, in particular involving the connectivity of the uncinate fasciculi [58]. Thus, in addition to disinhibition symptoms considered by Damasio et al., with evidence of potentially greater degree of WM rather than cortical injury, there is also similarity between Mr. Gage's behavioral changes and network alterations observed in FTD and related WM degenerative syndromes. This suggests that network topological changes may have been the source of Mr. Gage having not only executive function deficits but also problems resulting from damage to connections associated with the encoding of episodic memory as well as the processing of emotion – consistent with reports on changes in his personality.

### Historical Implications of Gage's WM Damage

While observations of severe network damage and their resulting affects may not be surprising given that which has been documented of Mr. Gage's accident and behavioral changes, one can only speculate upon the possible contribution to Gage's survival, recovery, and the uniqueness of changes to his WM networks. Macmillan [3] has noted that many reports on Gage's behavioral changes are anecdotal, largely in error, and that what we formally know of Mr. Gage's post-accident life comes largely from the follow-up report of Harlow [23] according to which Gage, despite the description of him having some early difficulties, appeared to adjust moderately well for someone experiencing such a profound injury. Indeed, the recent discovery of daguerreotype portraits of Mr. Gage show a “handsome…well dressed and confident, even proud” man [59] in the context of 19^th^ century portraiture. That he was any form of vagrant following his injury is belied by these remarkable images. While certainly neuroanatomically profound, the changes to his cognitive capacities were much more subtle upon his full recovery than may have been otherwise described. In spite of recovering from severe brain trauma, his mental state appears to have eventually stabilized sufficiently for him to travel throughout New England, take on several (some might say menial) forms of employment, travel through South America for several years, and to return to his family in the Western US, before succumbing to epilepsy which was presumably related to the injuries directly affecting his WM connectivity. That his network damage, though extensive, was not apparently more severe than an “average” brain lesion would incur may help to explain his ability to have sufficiently recovered in spite of the residual behavioral changes reported by Harlow.

### Limitations of our Study

We have worked to provide a detailed, accurate, and comprehensive picture of the extent of damage from this famous brain injury patient and its effect on network connectivity. While the approach used here to model the tamping iron's trajectory is precise and the computation of average volume lost across our population of subjects is reflective of the acute level of damage, we acknowledge that there was likely more damage than that caused by its presence alone. The iron likely propelled unrecovered bone fragments through the brain. The resulting hemorrhage from the wound was also considerable. Subsequent infection and a large abscess took further toll. Consequently, more GM and WM tissue may have been lost than estimated here. Like Damasio *et al.* and Ratiu *et al.*, we make the assumption that Gage's brain and its position within the skull can be estimated from the structure of the skull itself, and that its sub-regions, WM, and connective anatomy can be localized through population averaging. Such a supposition may have its limitations and could be open to debate. Nevertheless, ours represents the best current estimation as to the extent of brain damage likely to have occurred at the level of both cortex and WM fiber pathways. We also have no way of assessing the biochemical cascade of changes to biomarker proteins measureable post-injury in modern TBI patients which may also have influenced the trajectory of Mr. Gage's recovery.

Another potential criticism is that we compare the loss of GM, WM, and connectivity in Mr. Gage by computationally casting the tamping iron through the WM fibers of healthy age- and gender-matched subjects and measuring the resulting changes in network topology. We also systematically lesion the brains of our healthy cohort to derive “average” network metrics and compare the observed values with respect to them – an approach that has been recommended elsewhere [35]. This technique is helpful for creating a representative expectation of inter-regional connectivity against which to compare observed or hypothetical lesions. However, some might consider this approach to be misguided in this instance due to the fact that Mr. Gage's brain was damaged in such a way that he survived the injury whereas a host of other lesions resulting from penetrative missile wounds would likely have resulted in death. Indeed, as noted originally by Harlow, the trajectory of the 110 cm long, 3.2 cm thick, 13 lb. tamping iron was likely along the only path that it could have taken without killing Mr. Gage. Thus, any distribution of lesioned topological values might not provide a useful foundation for comparison because the majority of these penetrative lesions would, in reality, be fatal. We recognize these concerns and the practical implications for subject death which would also be a caveat of other network theoretical applications of targeted or random network lesioning. Indeed, such considerations are something to be taken into account generally in such investigations. Nevertheless, our simulations provide supporting evidence for the approximate neurological impact of the tamping iron on network architecture and form a useful basis for comparison beyond utilizing the intact connectivity of our normal sample in assessing WM connectivity damage. So, while this might be viewed as a limitation of our study, especially given the absence of the actual brain for direct inspection, the approach taken provides an appropriate and detailed assessment of the probable extent of network topological change. All the same, we look forward to further work by graph theoreticians to develop novel approaches for assessing the effects of lesioned brain networks.

### Conclusions

In as much as earlier examinations have focused exclusively on GM damage, the study of Phineas Gage's accident is also a study of the recovery from severe WM insult. Extensive loss of WM connectivity occurred intra- as well as inter-hemispherically, involving direct damage limited to the left cerebral hemisphere. Such damage is consistent with modern frontal lobe TBI patients involving diffuse axonal injury while also being analogous to some forms of degenerative WM disease known to result in profound behavioral change. Not surprisingly, structural alterations to network connectivity suggest major effects on Mr. Gage's overall network efficiency. Connections lost between left-frontal, left-temporal, right-frontal cortices as well as left limbic structures likely had considerable impact on executive as well as emotional functions. Consideration of WM damage and connectivity loss is, therefore, an essential consideration when interpreting and discussing this famous case study and its role in the history of neuroscience. While, finally, the quantification of connectomic change might well provide insights regarding the extent of damage and potential for clinical outcome in modern day brain trauma patients.

## Methods

### Ethics Statement

No new neuroimaging data was obtained in carrying out this study. All MRI data were drawn from the LONI Integrated Data Archive (IDA; http://ida.loni.ucla.edu) from large-scale projects in which subjects provided their informed written consent to project investigators in line with the Declaration of Helsinki, U.S. 45 CFR 46, and approval by local ethics committees at their respective universities and research centers. Research neuroimaging data sets deposited with the LONI IDA and made available to the public are fully anonymized with respect to all identifying labels and linked meta-data for the purposes of data sharing, re-use, and re-purposing. IDA curators do not maintain linked coding or keys to subject identity. Therefore, in accordance with the U.S. Health Insurance Portability and Accountability Act (HIPAA; http://www.hhs.gov/ocr/privacy), our study does not involve human subjects' materials.

### Medical Imaging of the Gage Skull

Medical imaging technology has been applied to the Gage skull on three known occasions to model the trajectory of the tamping iron, infer extent of GM damage, and theorize about the changes in personality which a patient with such an injury might have incurred. In an influential study, Damasio and coworkers [7] used 2D X-rays to obtain the dimensions of the skull itself and to compute the trajectory of the iron bar through the regions of frontal cortex based on independently obtained CT data from a normal subject. Prior to this, CT scanning of the skull had been obtained by Tyler and Tyler in 1982 for presentation and discussion at a neurological scientific meeting. The location of the raw CT data files from this imaging session is unknown but the data were last reproduced in *An Odd Kind of Fame* (Appendix E), though they were not part of any other scientific publication of which we are aware. The most recent occurrence of scanning on record was performed on June 12^th^, 2001 through the Surgical Planning Laboratory (SPL) at Brigham and Women's Hospital, Harvard Medical School. A series of two high-resolution CT image series were obtained of the skull: one covering the portion of the jaw up to approximately the bridge of the nose, and another covering the cranial vault (see details below). These data were used by Ratiu et al. [8], [60] to digitally reconstruct and animate the passage of the tamping iron through the skull. An additional CT image of the Gage life-mask, a plaster likeness presumed to have been commissioned by Dr. Bigelow during one of Gage's visits to Harvard Medical School, was also obtained and used to create a surface model of Mr. Gage's face, scalp, and neck. New CT or other medical imaging of the skull specimen is unlikely to be performed in the future due to the age and fragile state of the specimen.

### Documented Extent of Neurological Damage

In the book *An Odd Kind of Fame* (2000, pg 85), Macmillan conveniently summarizes the reports from various anatomists on the damage to Gage's brain. We reproduce these summaries here and also add the findings of Ratiu *et al.* [8] which appeared after the publication of *An Odd Kind of Fame*.

### Skull CT Data Processing

Due to a variety of circumstances, the raw and processed digital imaging data from the 2001 CT imaging session at Brigham and Women's Hospital were improperly archived and effectively lost to science. However, these image volumes were subsequently recovered by the authors and represent the highest quality data/resolution available (0.5 mm slice thickness) for modeling the skull of this noted patient and for use in the modeling of affected anatomy and connectivity. The scan data were originally obtained with the superior, cut portion of the calvarium and the mandible in the correct anatomical position on a Siemens Somatom CAT scanner (Siemens AG, Erlangen, Germany), in the Department of Radiology, Brigham and Women's Hospital (Boston, MA) [8]. These data were converted from ECAT format to the NIFTI file format (http://nifti.nimh.nih.gov) using the program “mri_convert” – part of the FreeSurfer neuroimaging data analysis software package (surfer.nmr.mgh.harvard.edu/fswiki/mri_convert). The CT images were systematically segmented and masked by hand using MRICron (http://www.cabiatl.com/mricro/mricron/index.html) and seg3D (http://www.sci.utah.edu/cibc/software/42-seg3d.html) to isolate the skull cap (the portion of the skull created by its being cut with a saw upon deposition at the Warren Museum by Dr. Harlow), each piece of remaining/healed bone fragments, the left frontal/temporal portion of the skull along the readily evident fracture lines, and the lower jaw, and separate 3-D surface mesh models were generated for each segment using 3D Slicer (http://www.slicer.org). An additional binary image volume was created by hand-filling the space of the cranium that contained Gage's brain. This volume represents a digital version of the standard endocast often used in the analysis of paleontological specimens [61], [62], [63]. Use of the Gage skull and life mask CT data is courtesy of the SPL and the Warren Anatomical Museum at Harvard Medical School.

### The LONI Pipeline Workflow Environment

For all major image processing operations (e.g. bias field correction, skull stripping, image alignment, etc.) we employed the LONI Pipeline Workflow Environment (http://pipeline.loni.ucla.edu; Fig. S1). This program is a graphical environment for construction, validation, and execution of advanced neuroimaging data analysis protocols. It enables automated data format conversion, leverages Grid computer systems, facilitates data provenance, and provides a significant library of computational tools [64], [65], [66].

For instance, employing LONI Pipeline, we used the Brainsfit software package (http://www.nitrc.org/projects/multimodereg/) to register the T1 anatomical MRI volumes to the endocast template. Diffusion gradient image data were processed in native subject space using Diffusion Toolkit (http://trackvis.org) to reconstruct the fiber tracts. Data processing workflows to compute inter-regional connectivity matrices were constructed using purpose-built software. Fig. S2 illustrates an example connectivity matrix displayed using Matlab (Mathworks, Natick, MA, USA).

### Measurements of the Skull

Consistent with Damasio *et al.*, the physical dimensions of the Gage skull were measured as follows in Table 2 using the Slicer software program. Additionally, the following landmarks were identified on the Gage skull: Entrance of the Left Auditory Canal: (49.56, 219.46, −807.75 mm); Entrance of the Right Auditory Canal: (175.04, 212.26, −802.85 mm); and the Middle of Crease Between Frontal Bone Plate and Nasal Bone: (117.04, 301.73, −800.72 mm). Given these landmarks, all the other points can be accurately positioned.

### Measurements of the Tamping Iron

One of our team (MCC) visited the Warren Anatomical Museum and, working with lead curator Dominic Hall, obtained the following measurements of the iron using a SPI Digimax caliper (Model: 30440-2): 110 cm in length, 9.5 cm circumference, and 2.88 cm diameter at tail. The rear taper is approximately 19 cm long, the maximum diameter (between the rear and tip taper) is 10.5 cm circumference (3.2 cm diameter), the taper beginning at the tip is 27 cm long, and the diameter at the rod's tip is 72 mm.

### The Trajectory of the Tamping Iron

The trajectory of the tamping iron through Mr. Gage's skull and brain has been the subject of much debate and several attempts have been made to infer the relationship between putative damage on the one hand and the lore surrounding Gage's personality and behavioral changes resulting from his accident on the other. Bigelow [67] first attempted to formally model the trajectory of the rod by drilling a hole through another “common” skull (pg. 21), and noted that “a considerable portion of the brain must have been carried away; that while a portion of its lateral substances may have remained intact, the whole central part of the anterior lobe, and the front of the sphenoidal or middle lobe must have been lacerated and destroyed”. Importantly, Damasio [7] and coworkers provided a detailed analysis of the rod trajectory through the skull attempting to identify which brain regions were impacted by the flight of the iron and what effect this impact had on the patient's post-injury behavior. While this study has been well cited, their methodology for determining the rod trajectory has been subsequently questioned [3].

Ratiu *et al.* [60] constrained their modeling of the rod trajectory by noting bony injuries to the skull, and by more closely aligning the rod with the clinical information provided by both Harlow and Bigelow. Ratiu *et al.* inserted the brain of a single normal subject into Mr. Gage's cranial cavity to examine which structures might have been affected. Their reconstruction shows that the path of the iron passed left of the superior sagittal sinus (their Fig. 4b,d). This is corroborated by the fact that damage to the superior sagittal sinus would have almost certainly caused air embolism and/or significant blood loss, resulting in Mr. Gage's death. In addition, their reconstruction shows, in their normal subject's brain, that the iron's trajectory was also anterior to the cingulate gyrus and to the left lateral ventricle (their Fig. 4 e,f). No rhinoliquorhea or other indication of post-traumatic CSF fistula was reported, nor that Gage developed ventriculitis, a condition which very likely would have been lethal - especially in the 1840's before the use of antibiotics in common medical practice. However, there is little way of being empirically precise with respect to location of major structures when employing only a single, example subject to represent Mr. Gage's unknown neuroanatomy.

To address this issue, we fit the T1 anatomical and diffusion images from the N = 110 normal, right handed subjects, aged 25–36 into the space of Phineas Gage's cranial vault to map the probability to regional injury and the effects of the tamping rod on WM fiber connectivity. The process of morphing data into the Gage skull is described in the following sections.

### Determining the Trajectory of the Tamping Iron

Using the measurements of the original tamping iron [3], [8], [24], [67], on display at the Warren Museum, a 3-D model of the tamping iron was generated using Matlab and stored as an VTK surface (http://www.vtk.org) for visualization using 3D Slicer and for processing using the segmented brain regions and fiber tracts morphed into the space of the Gage 3D cranial endocast volume model.

To constrain the trajectory of the rod through the Gage skull, we examined the work of previous authors to identify noteworthy statements on the condition of the skull, particular patterns of breakage, chips in the bone, and other prominent features that could be used as landmarks to restrict the possible paths which the rod might have taken (Fig. S3). For instance, the left maxillary molar is missing and osteological analysis by the Warren Museum states that it was lost *ante-mortem* (Object File WAM 00949, Warren Anatomical Museum, Francis A. Countway Library of Medicine). While Harlow and/or Bigelow do not specifically mention the loss of this tooth, it is likely that the rod made contact with it after passing through Gage's cheek, and was either dislodged completely or knocked loose and lost sometime during his recovery. Additionally, for the zygomatic arch the Warren Museum records (also WAM 00949) indicate “Maxilla: *ante-mortem* sharp force trauma remodeling” but are not more specific about the potential for complete breakage of the zygomatic process which was suspected by Ratiu *et al.* Still, it can be assumed that some contact was made between the iron and the interior portions of the arch. A collection of previously reported observations contributing to the set of applied constraints are noted in Table 3.

In particular, we concur with Ratiu *et al.* that Mr. Gage had his jaw open at the moment of the accident. Harlow reports Gage looking over his right shoulder and saying something to his crew at critical moment of the blast. In the casting of possible rod trajectories, the most likely position of the jaw was determined to be −15° in pitch (downward) and 5° in yaw (to the right) relative to the closed position of the jaw. This position allowed the unhindered passage of 1.303×10^3^ out of 1×10^9^ viable rod trajectories inclusive through the skull. With this jaw position, in contrast to the suspicion of Bigelow, we noted no contact between the rod and that of Mr. Gage's coronoid process. Jaw rotations at greater pitch angles were inconsequential to our results. Therefore, these values represent the minimal angular jaw deflections needed to allow the maximal number of rod passage scenarios without jaw intersection. Additionally, these values are typical for the acts of speaking and mastication in which the maximum typical jaw pitch extension in males is ∼30° [68]. Assuming the jaw to be in a completely closed position forces rod trajectories to incline more toward the right hemisphere in order to avoid contact with the jaw and breaking it - as may result from the trajectories identified by Damasio *et al.* Having the jaw open provides a greater number of possible paths which are closer to the vertical axis, which thus does not enforce an intersection of the rod with the right hemisphere (Fig. S4A, B, D; Fig. S5 A–D). The rod's intersection with white matter fiber tractography was thereby determined (Fig. S6). Movie S1 illustrates the path of the tamping iron through Mr. Gage's skull and the white matter fiber pathways of his left hemisphere.

### Normal Subjects

T1 anatomical MRI and 64-direction diffusion tensor images (DTI) from N = 110 right-handed male subjects between the ages of 25 and 36 were selected from the LONI Integrated Data Archive (IDA; http://ida.loni.ucla.edu). The age range was specifically selected to match the age at which Mr. Gage received his injury (25 years old) as well as the age at which he succumbed as a presumed result of the brain damage he experienced (36 years old). Subjects were all healthy “normals” with no neurological or history of psychiatric illnesses.

### Segmentation and Parcellation

Segmentation and regional parcellation were performed using FreeSurfer [69], [70], [71] following the nomenclature described in [72]. For each hemisphere, a total of 74 cortical structures were identified in addition to 7 subcortical structures and to the cerebellum. The 82 cortical and sub-cortical label names were assigned per hemisphere to each brain based upon the nomenclature described in Destrieux *et al.* [72]. Regional parcellation was performed using FreeSurfer [73], [74], [75], [76] (see also above). The numbers of hemispheric partitions in the segmentation was as follows – frontal (21), insula (8), limbic (8), temporal (12), parietal (11), occipital (14), basal ganglia (8), and brain stem (1). The complete coding scheme is as presented describing the parcellation scheme naming convention (Table 6) and their abbreviations (Table 7), which can be used to identify the regional labels in Figs. 2a and 3.

### Connectogram Design

Neuroanatomical structure and connectivity information were graphically depicted in a circular diagram format using freely available Circos software ([77], www.cpan.org/ports). Briefly, Circos is a cross-platform Perl-based application which employs a circular layout to facilitate the representation of relationships between pairs of positions by the use of various graphical elements, including links and heat maps. While traditionally used to render genomic information, Circos can be effectively adapted to the exploration of data sets involving complex relationships between large numbers of factors. In our case, cortical parcellations were represented as a circular array of 165 radially aligned elements representing the left and right cerebral hemispheres, each positioned symmetrically with respect to the vertical axis. We term this representation a “connectogram”. The brain stem was positioned at the most inferior extremity of the Circos ring as a consequence of its inclusion as the only midline structure. In this manner, Circos' ability to illustrate chromosomes was modified for lobar depiction, while its functionality for illustrating cytogenetic bands was modified to represent cortical parcellations. As previously described, each parcellation was assigned an arbitrary but unique RGB color (see below). Parcellations were arranged within each lobe in the order of their location along the antero-posterior axis of the cortical surface associated with the published FreeSurfer normal population atlas [72]. To determine this ordering, the center of mass was computed for the GM surface portion associated with each parcellation, and the order of all parcellations was determined based on the locations of these centers of mass as their distance from the frontal pole increased along the antero-posterior coordinate axis. A LONI Pipeline workflow for the creation of the connectogram images using parcellation and connectivity matrix information is available upon request from the authors. A complete description of the methods for connectogram construction can be found in [78] with applied examples in [79].

### Color Coding Schemes

Each cortical lobe was assigned a unique color scheme: black to red to yellow (Fro), charlotte to turquoise to forest green (Ins), primrose to lavender rose (Lim), pink to lavender to rosebud cherry (Tem), lime to forest green (Par), and lilac to indigo (Occ). Each structure was assigned its unique RGB color based on esthetic considerations; e.g. subcortical structures were colored light gray to black. Color scheme choice and assignment to each lobe were made by taking into account the arrangement and adjacency of lobes on the cortical surface, with the goal of avoiding any two adjacent lobes from having overlapping or similar color schemes which were too similar. The individual colors of the scheme associated with any particular lobe were assigned to every parcellation within that lobe in such a way as to create a distinct contrast when displayed on cortical surfaces (Fig. S2) or on the connectogram graphics (Figs. 2 and 3). The particular regional color mappings employed in this article can be considered arbitrary and are not intended to convey any universal or standard regional color scheme, *per se*.

### Representation of Cortical Metrics

Within the circular framework representing the cortical parcellations, five circular heat maps were generated, each encoding one of five structural measures associated with the corresponding parcellation. Proceeding inward towards the center of the circle in Fig. 2, these measures were: total GM volume, total area of the surface associated with the GM-WM interface (forming the base of the cortical ribbon), mean cortical thickness, mean curvature and connectivity per unit volume. For subject-level analysis, these measures were computed over the entire volumetric (or areal, as appropriate) extent of each parcellation; for the population-level analysis, they were averaged over all subjects.

Values for each measure were mapped to colors, using a scheme that ranged from the minimum to the maximum of the data set. For example, the cortical thickness *t* with values ranging from *t_min_* to *t_max_* was normalized as *t_1_* = (*t*−*t_min_*)/(*t_max_*−*t_min_*). The latter value was mapped onto a unique color from the color map of choice. Thus, for example, hues at color map extremities correspond to *t_min_* and *t_max_*, as required. For subcortical structures, brain stem and cerebellum, three measures (area, thickness and curvature) were unavailable on a parcellation-by-parcellation basis; their corresponding heat map entries were consequently left blank.

The connectogram in Fig. 3, illustrating the effects of the tamping iron lesion, represents the individual regionally-specific network metrics (i.e. betweenness centrality, eccentricity, mean local efficiency, and clustering coefficient) and are colored distinctly to be consistent with the cortical maps of the same but unaffected network metrics presented in Fig. 5A. The inner-most ring of the connectogram in Fig. 3 represents the average proportion of regional GM loss taken across subjects.

### Connectivity Calculation

To compute connectivity between regions for each subject, the location of each fiber tract extremity within the brain was identified, while the GM volume associated with each parcellation was also delineated. For those fibers which both originated as well as terminated within any two distinct parcellations of the 165 available, each fiber extremity was associated with the appropriate parcellation. For each such fiber, the corresponding entry in the connectivity matrix (e.g. Fig. S2) of the subject's brain was appropriately updated to reflect an increment in fiber count [80], [81]. Each subject's connectivity matrix was normalized over the total number of fibers within that subject; for population-level analysis, all connectivity matrices were pooled across subjects and averaged to compute probabilistic connection probabilities.

### Connectivity Representation

For subject-level connectograms, links were generated between any two parcellations whenever a WM tract existed between them. In population-level analyses, the former was done whenever there was a non-vanishing probability for a WM tract to exist between the two regions (Fig. 2). Links were color-coded by the average fractional anisotropy (FA) value associated with the fibers between the two regions connected by the link, as follows. The lowest and highest FA values over all links (*FA_min_* and *FA_max_*, respectively) were first computed. For any given connection *i* where *i* = 1, …, *N* (*N* being the total number of connections), the FA value *FA_i_* associated with that connection was normalized as *FA′_i_* = (*FA_i_*−*FA_min_*)/(*FA_max_*−*FA_min_*), where the prime indicates the *FA_i_* value after normalization. After this normalization, *FA′_i_* values were distributed in the interval 0 to 1, where 0 corresponds to *FA_min_* and 1 corresponds to *FA_max_*. The interval 0 to 1 was then divided into three subintervals (bins) of equal size, namely 0 to 1/3, 1/3 to 2/3, and 2/3 to 1. For every *i* = 1, …, *N*, link *i* was color-coded in either blue, green or red, depending on whether its associated *FA′_i_* value belonged to the first, second, or third bin above, respectively. Thus, these bins represent low, medium, and high FA. In addition to encoding FA in the link's color as described, relative fiber density (the proportion of fibers for each connection out of the total number of fibers) was also encoded as link transparency. Thus, within each of the three FA bins described, the link associated with the highest fiber density within that bin was rendered as completely opaque, whereas the link with the lowest fiber density was colored as transparent as possible without rendering it invisible. For example, the link with *FA′_i_* = 1/3 was colored as opaque blue, whereas the link with the lowest *FA′_i_* value was colored as most transparent blue. Similarly, the link with *FA′_i_* = 2/3 was colored as opaque green, and the link with the lowest value of *FA′_i_* greater than 1/3 was colored as faintest green. The links associated with the lowest fiber densities were drawn first, and links with progressively larger relative fiber densities were drawn on top of the former. The process was successively repeated by drawing links with higher fiber densities on top of links with lower fiber densities. Thus, links associated with the largest fiber densities were drawn “on top” of all other links.

### Representation of Connectivity Affected by Pathology

Links associated with fibers affected by pathology were designed to encode fiber density using the same transparency coding scheme as described in the previous subsection. In contrast with the case of healthy fibers, however, two different color schemes were used to encode pathology. Whenever fibers existed between one cortical region that was affected by pathology and another that was not, the color used to draw the corresponding link was brown. By contrast, links between parcellations that were *both* affected by pathology were drawn using the color gray. This allows one to visually distinguish between connections that involve only one affected region (brown links) and connections that involve two regions that were both affected (grayscale links) (Fig. 3).

### Calculation of Pathology Effects upon GM/WM Volumetrics

To compute the amount of GM/WM affected by pathology, two types of volume quantities were first computed. The first of these consisted of the individual volume 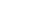 of each GM parcellated region 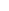 assuming a healthy brain, hence the dependence of 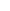 upon 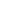. The second computed quantity was the spatial extent and total amount of brain volume that had been directly affected by the tamping iron. After identification of these volumes, the spatial intersection of the latter with each parcellation 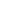 was computed. In other words, the pathology-affected portion of each parcellation, call it 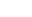, was identified by computing the intersection between that parcellation and the whole injury. From these intersections, the percentage of each parcellation that had been affected by pathology was obtained for each subject, and the means and standard deviations of these percentages were calculated over all subjects for each parcellation (Table 4).

The calculation described above estimated the amount of GM that was *directly* affected by the passage of the rod. To compute the total amount of GM that was affected by pathology, however, it is not sufficient to compute the sum of *directly* lesioned GM parcellation volumes because pathology-affected GM includes cells with intact somas whose axons were nevertheless injured in at least one location along their paths. In other words, a population of neurons whose GM axons were destroyed or affected in spite of their somas being outside the volume of direct injury should also be taken into account when computing the amount of affected GM. Furthermore, the destruction of fibers originating in some parcellated region *r_1_* that had been *directly* affected by pathology could also have affected the GM in parcellations to which *r_1_* is connected by WM fibers originating in *r_1_*. Consequently, an appropriate calculation of the total GM volume affected by pathology must take into account available quantitative information concerning the extent to which WM fibers affected by pathology could indirectly affect GM as well. To obtain and interpret such information meaningfully, one can use the measures of GM and WM atrophy described below:

Let *c_ij_* (*h*) be the probabilistic count of fibers between parcellated regions *r_i_* and *r_j_*, as computed over all healthy subjects using the methods described in the section on *Connectivity Calculation*. Note that *c_ij_* (*h*) is the connectivity matrix entry which specifies, in a probabilistic sense, the proportion of fibers between parcellated regions *r_i_* and *r_j_*. The dependence of the count *c_ij_* upon the parameter *h* (denoting health) reflects the fact that the fiber density can be different depending on whether the parcellated region has or has not been affected by pathology. For the former scenario, the count is denoted by *c_ij_* (*p*), where *p* stands for pathology. If two parcellations *r_i_* and *r_j_*, are unaffected, then 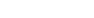
If, however, either one or both of *r_i_* and *r_j_* are affected, then 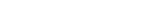
where *c_ij_* (*d*) stands for the count of fibers that were destroyed (hence *d* as the argument) as a result of the injury. The change in fiber count from health to pathology between two regions untouched by the rod reflects the extent to which the somas of the neurons connecting the regions have been affected by direct injuries to the WM fibers between them. Consequently, it is reasonable to posit that an appropriate measure of GM injury in this case can be formulated by relating the proportion of destroyed WM fibers between two regions to the proportion of affected GM volume within the regions. For this purpose, we computed the metric
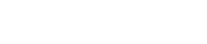
For regions *r_i_* and *r_j_*, the ratio in square brackets on the left hand side (LHS) of the equality above is the proportion of fibers connecting the two regions that are affected by pathology (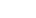), out of the total number of healthy fibers 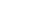 between the two regions, where the latter value is computed probabilistically as already described. Note that
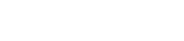
as expected. The second ratio on the LHS of the equality defining 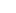 is the sum of the volumes of GM regions *r_i_* and *r_j_* as computed in the absence of the injury (hence their explicit dependence upon *h*), normalized by the total volume of all *M* regions. In other words, this second ratio is the proportion of the healthy brain's GM associated with regions *r_i_* and *r_j_*. An observation that is essential for grasping the physical meaning of 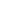 is that, because each neuron in *r_i_* can be directly connected to *r_j_* via no more than one axon, the fraction of fibers affected by pathology—i.e. 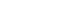
—is directly proportional to the fraction of the combined volume of the regions *r_i_* and *r_j_* that was affected. In other words, 
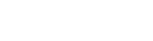
Histological research [82] (and references therein) indicates that the constant of proportionality can be assumed to be approximately equal to 1, i.e. 
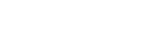
This relationship is useful because, whereas calculation of the ratio on the LHS is straightforward from DTI tractography as previously described, that of the ratio on the RHS is not because regions *r_i_* and *r_j_* do not necessarily intersect the passing rod.

With the assumptions above, it follows that the quantity 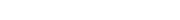 can be thought of as the fraction of GM volume in regions *r_i_* and *r_j_* that was affected by direct injury to WM only, while the metric 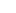 corresponds to the affected GM volume in *r_i_* and *r_j_* as a fraction of the total GM volume of the brain, the latter being the summation in the denominator of 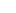.

A second metric of interest is the ratio
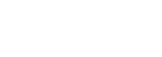
where 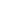 denotes the arithmetic average operator. The numerator 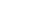 is the mean length of the fibers between regions *r_i_* and *r_j_*, as computed from DTI tractography using methods already described. The denominator is simply the sum of fiber lengths between all regions, and can be thought of as the total length of WM “wiring” in the brain. The purpose of the restriction *i<j* imposed upon the summation is to ensure that (1) no fibers are counted twice, and that (2) no fibers between cortical columns within the same region (i.e. *i* = *j*) are included in the summation. In summary, the metric above represents the fraction of the length of WM fibers between regions *r_i_* and *r_j_* to the sum of fiber lengths in the entire brain. Consequently, it can be thought of as a measure of the fraction of WM associated with the two regions out of the total amount of WM available. It should be noted that, as expressed above, 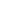 can depend upon either *p* or *h*, depending on whether the fiber whose length is denoted by 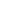 has been affected by pathology or not, respectively. The denominator of the fraction, however, depends on *h* alone because it is the sum of fibers in the unaffected brain. It should be noted that the denominator of equation for 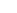 (i.e. the total GM volume of the healthy brain) is distinct from that of 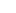 (i.e. the total WM volume of the healthy brain). The numerator, on the other hand, can refer to the mean length of a fiber which may or may not have been destroyed, as necessary. Specifically, 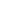 can be equal to 0 (if the fiber indexed by *i* and *j* in the connectivity matrix was destroyed), or to 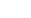 (i.e. the length of the fiber in the healthy brain, if that fiber was not affected).

In summary, the metrics 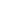 and 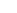 describe the respective proportions of GM and WM that were affected by pathology as a fraction of the total GM or WM volume of the brain, respectively. The marginal probability density functions of 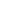 and 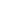 were also computed and displayed Fig. 4A and 4B. The metrics 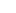 and 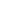 from all parcellation pairings themselves may be plotted as a bivariate probability density function as in Fig. 4C. In the case of Mr. Gage, this plot illustrates that, unsurprisingly, while a large amount of relatively short, local fibers were lost due to the penetration of the tamping iron, there were additionally a range of longer and denser fiber pathways between distributed brain areas which were also affected.

### Average Percentages of Brain Regions Intersected by the Rod

The average percentage regional volumes (and their standard deviations) intersected by the rod pooled over N = 110 subjects are listed in Table 7 and illustrated graphically in the connectogram of Fig. 3.

### Network Analysis

Because network theory can provide essential insight into the structural properties of cortical connectivity networks in both health and disease [83], several network metrics of particular significance were computed for each subject, starting with the *degree* of each node. In our case, nodes were denoted by parcellated regions and edges were represented by fiber tracts. Nodal degree is the number of edges connected to a node and its calculation has fundamental impact upon many network measures; moreover, node degree distributions are highly informative of network architecture. The entry indexed by *i* and *j* in the *distance* matrix of the graph contains the minimum weighted physical length of the path connecting vertices *i* and *j* and was computed using the algebraic shortest paths algorithm [84]. Degree of connectivity is represented as the inner-most ring in Fig. 2, though was not analyzed further beyond its being utilized in the computations of some of the overall network metrics detailed below.

The measurement of network attributes can be generally broken down into the examination of overall network *integration* – the measurement of path lengths between nodes in a network and the extent of network-wide interaction and ease of communication between distinct regions; *segregation* – the extent to which nodes of the network group themselves into separate communities; and *small worldness* – the quantification of the generally shorter path lengths and higher clustering observed in many biological and technological networks with respect to randomly connected systems [85]. To specifically measure these overall network properties, we chose to focus on three particular metrics. To assess network integration from each subject's connectivity matrix we measured the characteristic path length, a measurement of the global average of a graph's distance matrix [86]. Appropriate to our application, the weighted characteristic path length of a network may be altered as a result of brain trauma [87]. To measure the degree of segregation, we computed the *mean local efficiency* of each network. Investigating network segregation can be important because it can reveal how much information brain regions are able to exchange as well as the extent to which such regions remain structurally segregated from each other. In this instance, reduced efficiency might be expected as a result of a severe penetrating head wound. Finally, we measured network *small worldness*, i.e. the ratio comprised of the observed characteristic path length relative to that observed in a random network having the same degree distribution and the observed clustering coefficient relative to that observed in a random network.

Additionally, to characterize the regionally-specific effects of the tamping iron lesion, we also computed several additional graph theoretical measurements for each parcellated brain region. These included 1) betweenness centrality, measuring the number of shortest paths from all vertices to all others that pass through that node, 2) local efficiency, the mean shortest absolute path length of at that node, 3) clustering coefficient, measuring the degree to which a node is nodes in a graph is a member of a cluster or clique, and 4) eccentricity, representing the greatest geodesic distance between that node and any other vertex in the graph. Metrics were computed for each subject and averaged with respect to weighting by subject-wise regional parcellation volume. To be consistent with other studies reporting these regionally-specific values, we chose not to normalize them with respect to those obtained in equivalent random networks. Averages of these metrics are illustrated in Fig. 5a(i–iv) along with linear colorbars indicating the ranges of observed mean values. Effects on these metrics in the presence of the tamping iron can be seen as the first four of the inner-most rings of the connectogram presented in Fig. 3.

Several additional global as well as local graph metrics were computed but not reported here due to potentially excessive colinearlity, imprecision, or due to recognized difficulty with interpretation. For instance, network *modularity* [88] was not considered due to the heuristic nature of its computation and tendency to provide unreliable values upon repeated estimation. While many of these other network metrics are well known and have their unique advantages [83], the ones chosen parsimoniously capture the overall changes in network architecture for this patient and the extent to which his injury would compare to similarly-sized lesions in other areas of the cortex. The Brain Connectivity Toolbox (BCT; https://sites.google.com/a/brain-connectivity-toolbox.net/bct/Home) was used for all weighted and unweighted connection density- and path-length related graph theoretical computations [84].

For each of the global graph theory measures described above, the mean and standard deviation was computed for each subject in both intact (healthy) and pathology-affected scenarios (the tamping iron lesion as well as simulated lesions over the brain). As an additional basis, we also performed a degree-preserving randomization process using the BCT for each subject's intact network, computed the aforementioned network measurements, and report these averaged across subjects. Such normalization has been recently advised by Rubinov and Sporns [84]. In our case, this involved 10,000 “rewiring” iterations of the BCT *null_model_und_sign* (compiled C-code version of the Matlab code from the “the bct-cpp project”; http://code.google.com/p/bct-cpp) algorithm per region by subject. To accommodate the computational cost of performing such a randomization process, we utilized fully the 1200 node Linux cluster based at the Laboratory of Neuro Imaging (LONI) at UCLA to randomize subjects and regions in parallel. Incidentally, normalization of each network type by its own randomized version has the effect of scaling out differences between networks – lesioned or otherwise – and thus makes the metrics largely insensitive to the effects of network damage. So, to provide a common frame of reference across each network type, the observed metrics for the intact, tamping iron, and simulated lesions were normalized with respect to the degree-preserving randomization of the intact network. Finally, to specifically test the differences between the intact and the tamping iron-lesioned networks between subjects, paired Student's t-tests were applied for each normalized measure to identify significant differences between means at p≤0.01. Results are summarized in Table 5. Further details on the lesion simulation are provided in the section below.

### Equivalent Lesion Simulation and Comparison

To examine the tamping iron lesion's specificity to changes in network structure, we investigated whether changes Gage's brain network properties were significantly different from those that would be expected by chance for the same amount of GM loss located in other regions of the brain. To address this, network properties were computed for a set of simulated lesions systematically positioned over the cortex (excluding the tamping iron lesion itself) and Mr. Gage's network measurements were compared to the distribution of the average metric values taken over subjects and lesions. Specifically, we adopted an approach similar to that of Alstott *et al.* [89], who simulated the effects on functional connectivity of targeted lesions distributed in various regions of the cerebral cortex. In our extension of this method, localized area removal was performed by deleting all nodes and their connections within regions consisting of contiguous anatomic parcellations as defined using the methods of Destrieux et al. [72]. In contrast to Alstott et al., however, our structural connectivity simulations also sought to account for additional lesion effects upon WM by modeling the removal of so-called “fibers of passage”. To do so, connectivity network edges between anatomic parcellations neighboring the GM lesion were removed without deleting the corresponding nodes connected by these edges, unless these nodes also belonged to the GM portion of the lesion itself.

The details of our simulation are as follows: 500 distinct lesions were simulated by first populating the cortical surface with 500 distinct sets of contiguous parcellations. Each of these sets was subsequently used as a synthetic “lesion”, subject to the constraints that the percentages of WM and GM lost due to the lesion were the same as had been estimated for Gage's tamping iron injury. This process was repeated until 500 distinct lesions were created uniformly across the brain, and the procedure was repeated for all 110 subjects included in the study. To ensure that each of the lesions had approximately the same position in each subject, lesion configurations were defined using the cortical atlas of Fischl, Dale et al. [71], and the corresponding location of every lesion in each subjects was identified by mapping the lesion configuration from the atlas to each subject's cortical surface using existing/published FreeSurfer methodology [70], [90], [91]. Thus, by the process described above, 500 distinct lesions that were identical in size to Gage's from the standpoint of percentage WM and GM loss were created uniformly over the brain in each of the 110 subjects. Subsequently, each lesion's effect on overall network properties was computed. Global network metrics were then pooled over all subjects and simulations so as to obtain the average (i.e. most probable) value of every metric for each of the 500 simulated lesioned networks.

In this context, for each network metric, the null hypothesis was formulated as the statement that the metric value associated with Gage's lesion of left frontal cortex was drawn from the same distribution as that of the “average” cortical lesion. This comparison of changes in network properties as a function of lesion location is one viable and interesting way to assess whether Mr. Gage's brain network properties were significantly different from those that would be expected by chance for the same amount of GM and WM loss. Specifically, for each metric *m*, the whole brain mean *μ*(*m*) and standard deviation *σ*(*m*) of the metric was first computed over lesions. Subsequently, for the metric value *m_T_* associated with each lesion, the standard score 
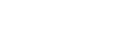
was computed. Results for the average properties in the intact networks, the tamping iron injury, and the lesion simulations – in addition to their degree-preserved randomized comparison versions - are illustrated in Fig. 5b (i and ii). Similar calculations and comparisons on the basis of small worldness provided patterns highly similar to that for network integration, thus were deemed redundant, and therefore are not illustrated here.

Finally, we compared the observed effects of the tamping iron lesion on the random network normalized graph theory measures of integration and segregation against that observed for all remaining lesions. Computed as Z-statistics, the results of these comparisons are illustrated graphically for network integration and segregation in Fig. 5c (i and ii), respectively, and are colored to show those effects most similar to the tamping iron lesion (black), moderately similar (orange), and most dissimilar (white). Generally, as one moves posteriorly away from the Gage lesion site, similarity on network effects tends to be reduced. However, exceptions exist in bilateral post-central gyrus and the left superior and posterior portion of the parahippocampal gyrus.

## Supporting Information

Figure S1 **The LONI Pipeline Workflow Environment.** We applied the LONI Pipeline [93], [94] for segmentation and registration of the input MRI image volume data, the processing of all DTI tractography, and computation of tract statistics. This grid-based solution provides validation and distribution of new computational tools, and an intuitive graphical interface for developing and executing parallel volumetric processing software. See http://pipeline.loni.ucla.edu for additional details.
(TIF)

Figure S2 **Views of the cortical parcellation of a sample subject.** Top rows show the lateral, anterior, and dorsal surfaces; second row shows medial, posterior, and ventral pial surfaces, while the bottom two rows show the same orientations but as inflated pial surfaces to more adequately present the extent of regional parcellations and their color coding. The arbitrarily chosen regional colors are the same as those of the outer-most ring in Figure 2 and whose RGB values are referenced Table 5 are shared by the outer most ring of brain regions on the connectogram images permitting rapid cross-reference.
(TIF)

Figure S3 **Connectivity Matrix.** Each row and each column represent distinct parcellated regions where in each cell i,j was computed the number of fibers that were found to begin or end in each region pair, the average FA, and the average fiber length over subjects.
(TIF)

Figure S4 **Modeling of the Skull Fragmentation and the Rod.** a) Models of the eyeballs were placed in to the ocular cavities in order to use them as constraints for the trajectory of the tamping iron. According to Harlow's account, the left orbit was extended outward “by half its diameter”. b) The bones of the skull representing the major breakages were systematically labeled and can be independently manipulated using Slicer. The mandible was also rotated downward and laterally in order to allow the tamping iron not to impinge on it and also to comply with Harlow's account that Gage was in the act of speaking to his men at the moment of the blast. c) The surface model of the Gage skull, with closed mandible, along with the surface of the life mask commissioned by Bigelow. d) A view looking superiorly along the tamping iron's computed trajectory noting how the iron displaced the left anterior frontal bone as it passed.
(TIF)

Figure S5 **Illustrating the Intersection of the Rod and the Brain.** a) A figure showing the passage of the rod through the skull with the bones above the cranial “cap” cut at Harlow's direction, and its intersection with the left anterior white matter fiber pathways of an example subject. The complementary hemisphere is displayed to illustrate that the rod did not intersect that hemisphere. b) A view of the rod displacing the bones of the skull. c) A close up, coxial view of the inferior portion of the iron along its trajectory. d) The intersection of the tamping iron with the left frontal cortex with each major bone fragment removed.
(TIF)

Figure S6 **The Effects of the Tamping Iron on White Matter Fiber Tractography.** a) A view of the Gage skull with the white matter fiber tracts of an example subject warped to the space. In this view, fibers which intersect the rod's pathway have been removed. b) A transaxial view of the DTI fiber pathways remaining after those which were intersected by the rod had been removed. c) The fibers intersected by the rod connect areas of cortex throughout the left cerebral hemisphere as well as between hemispheres. d) A sagittal view of the fibers experiencing damage by the tamping iron. All bone fragments and the cranial “cap” have been removed.
(TIF)

Movie S1 **Movie of The Effects of the Tamping Iron on White Matter Fiber Tractography.** This movie rendering illustrates the passage of the tamping iron through the Gage skull and its intersection with left hemispheric white matter fiber pathways. The right hemispheric cortical surface model is displayed to illustrate that the rod did not cross the midline to damage right frontal cortex. The rendering was created using 3D Slicer (http://slicer.org).
(WMV)
