# Supplementary figures and images for: Mapping Connectivity Damage in the Case of Phineas Gage
Source: PLoS One. 2012 May 16;7(5):e37454. doi: 10.1371/journal.pone.0037454 (PMC3353935; doi:10.1371/journal.pone.0037454)

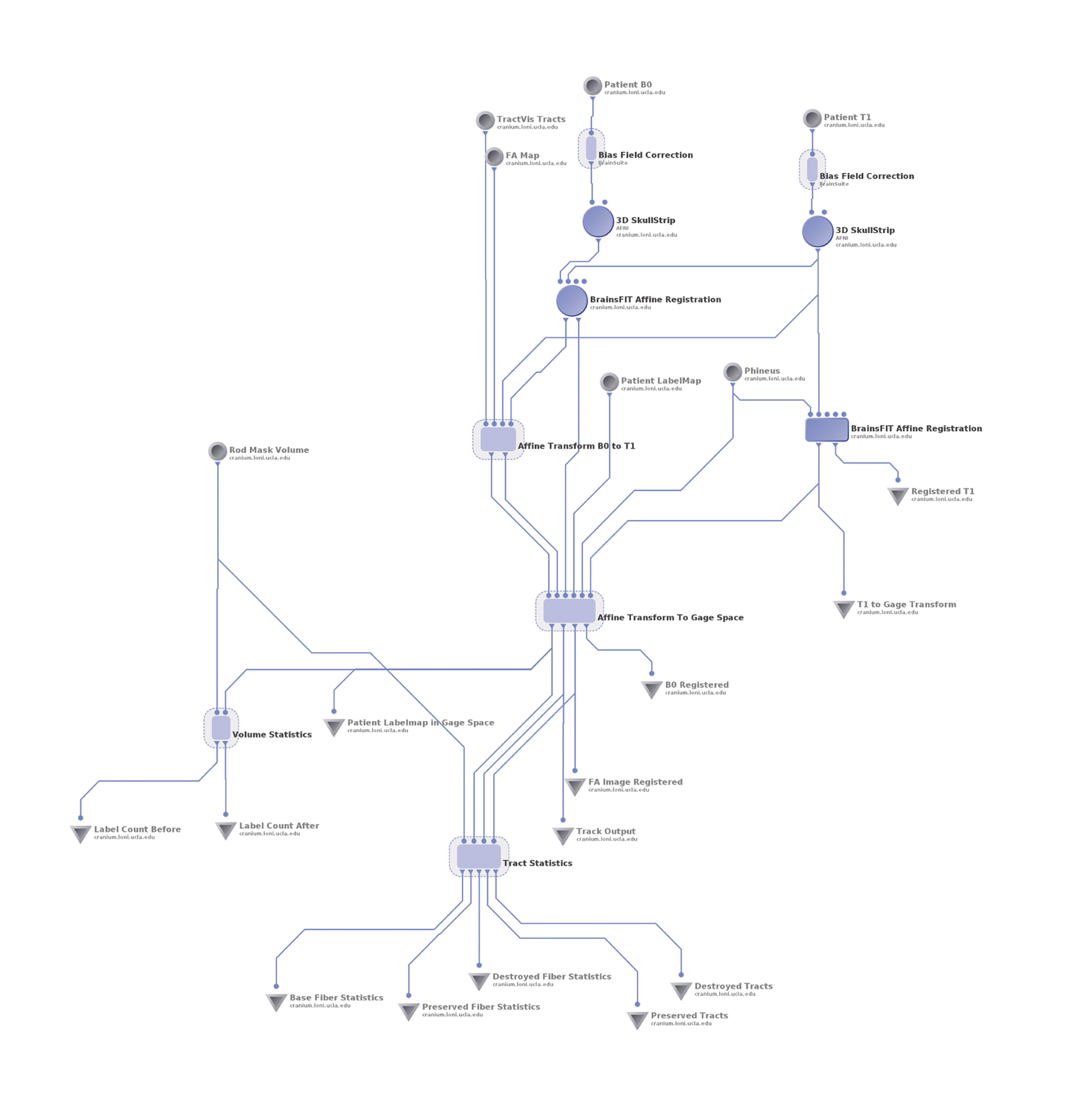

Supplement: Figure S1 — The LONI Pipeline Workflow Environment. We applied the LONI Pipeline [93], [94] for segmentation and registration of the input MRI image volume data, the processing of all DTI tractography, and computation of tract statistics. This grid-based solution provides validation and distribution of new computational tools, and an intuitive graphical interface for developing and executing parallel volumetric processing software. See http://pipeline.loni.ucla.edu for additional details. (TIF) [file pone.0037454.s001.tif]

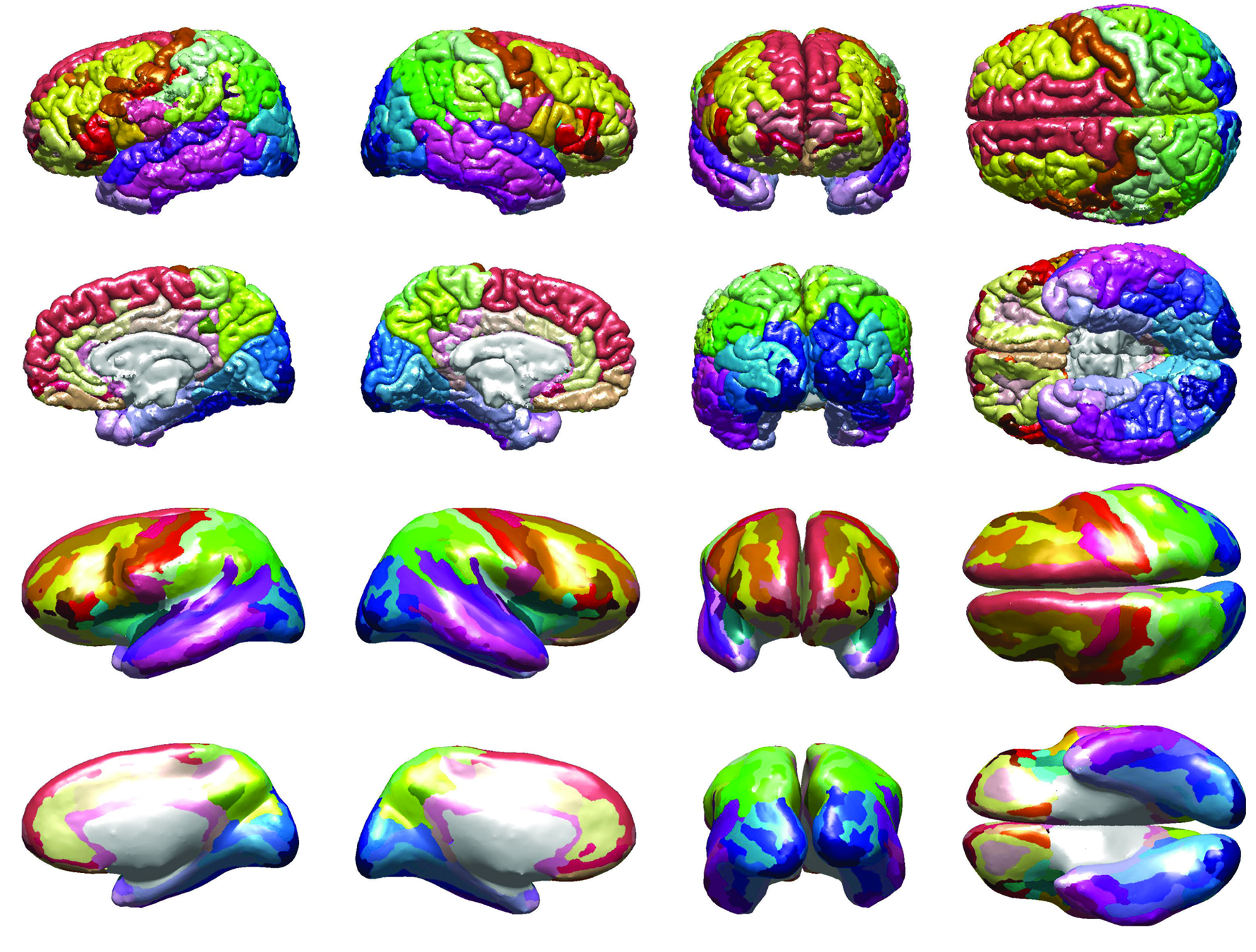

Supplement: Figure S2 — Views of the cortical parcellation of a sample subject. Top rows show the lateral, anterior, and dorsal surfaces; second row shows medial, posterior, and ventral pial surfaces, while the bottom two rows show the same orientations but as inflated pial surfaces to more adequately present the extent of regional parcellations and their color coding. The arbitrarily chosen regional colors are the same as those of the outer-most ring in Figure 2 and whose RGB values are referenced Table 5 are shared by the outer most ring of brain regions on the connectogram images permitting rapid cross-reference. (TIF) [file pone.0037454.s002.tif]

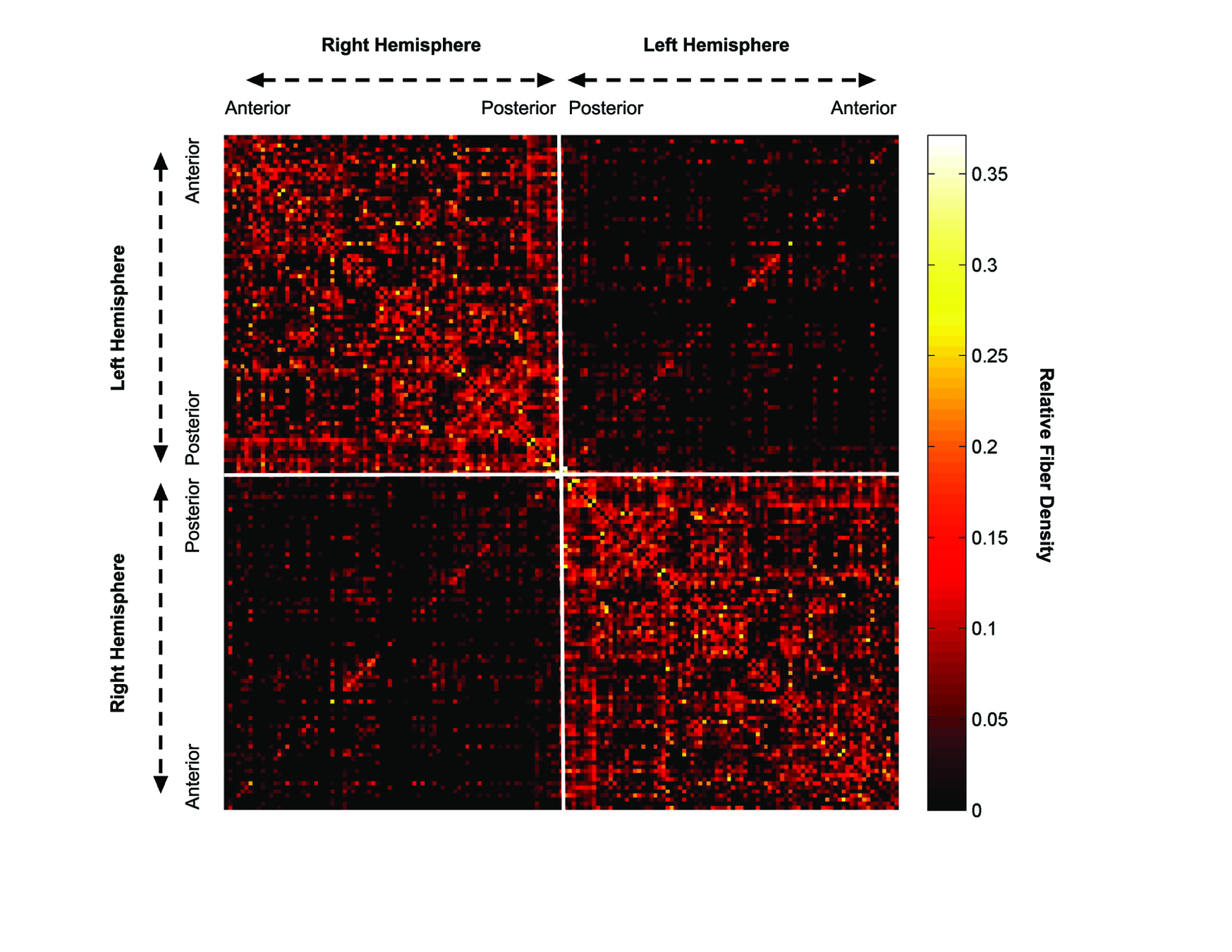

Supplement: Figure S3 — Connectivity Matrix. Each row and each column represent distinct parcellated regions where in each cell i,j was computed the number of fibers that were found to begin or end in each region pair, the average FA, and the average fiber length over subjects. (TIF) [file pone.0037454.s003.tif]

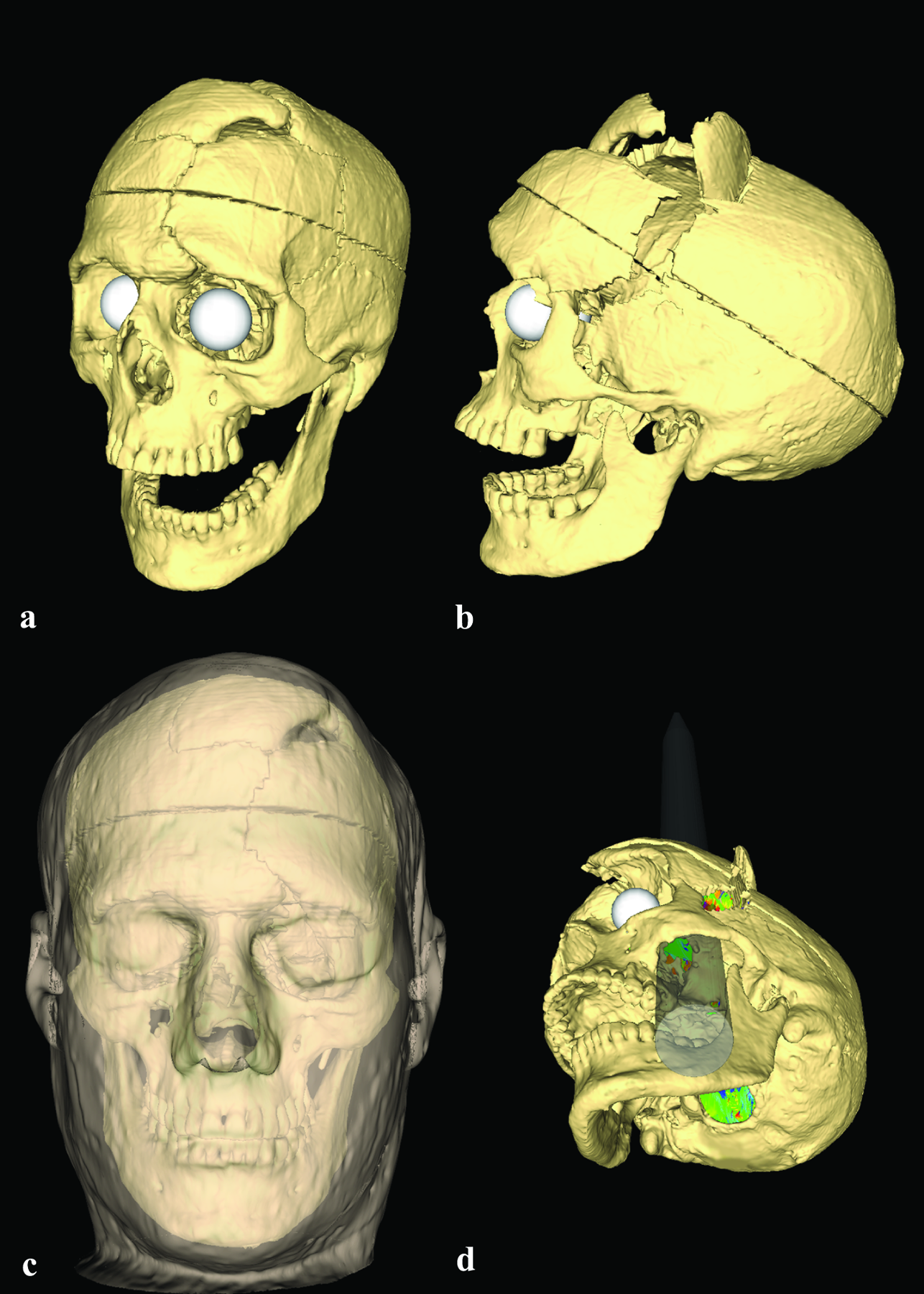

Supplement: Figure S4 — Modeling of the Skull Fragmentation and the Rod. a) Models of the eyeballs were placed in to the ocular cavities in order to use them as constraints for the trajectory of the tamping iron. According to Harlow's account, the left orbit was extended outward “by half its diameter”. b) The bones of the skull representing the major breakages were systematically labeled and can be independently manipulated using Slicer. The mandible was also rotated downward and laterally in order to allow the tamping iron not to impinge on it and also to comply with Harlow's account that Gage was in the act of speaking to his men at the moment of the blast. c) The surface model of the Gage skull, with closed mandible, along with the surface of the life mask commissioned by Bigelow. d) A view looking superiorly along the tamping iron's computed trajectory noting how the iron displaced the left anterior frontal bone as it passed. (TIF) [file pone.0037454.s004.tif]

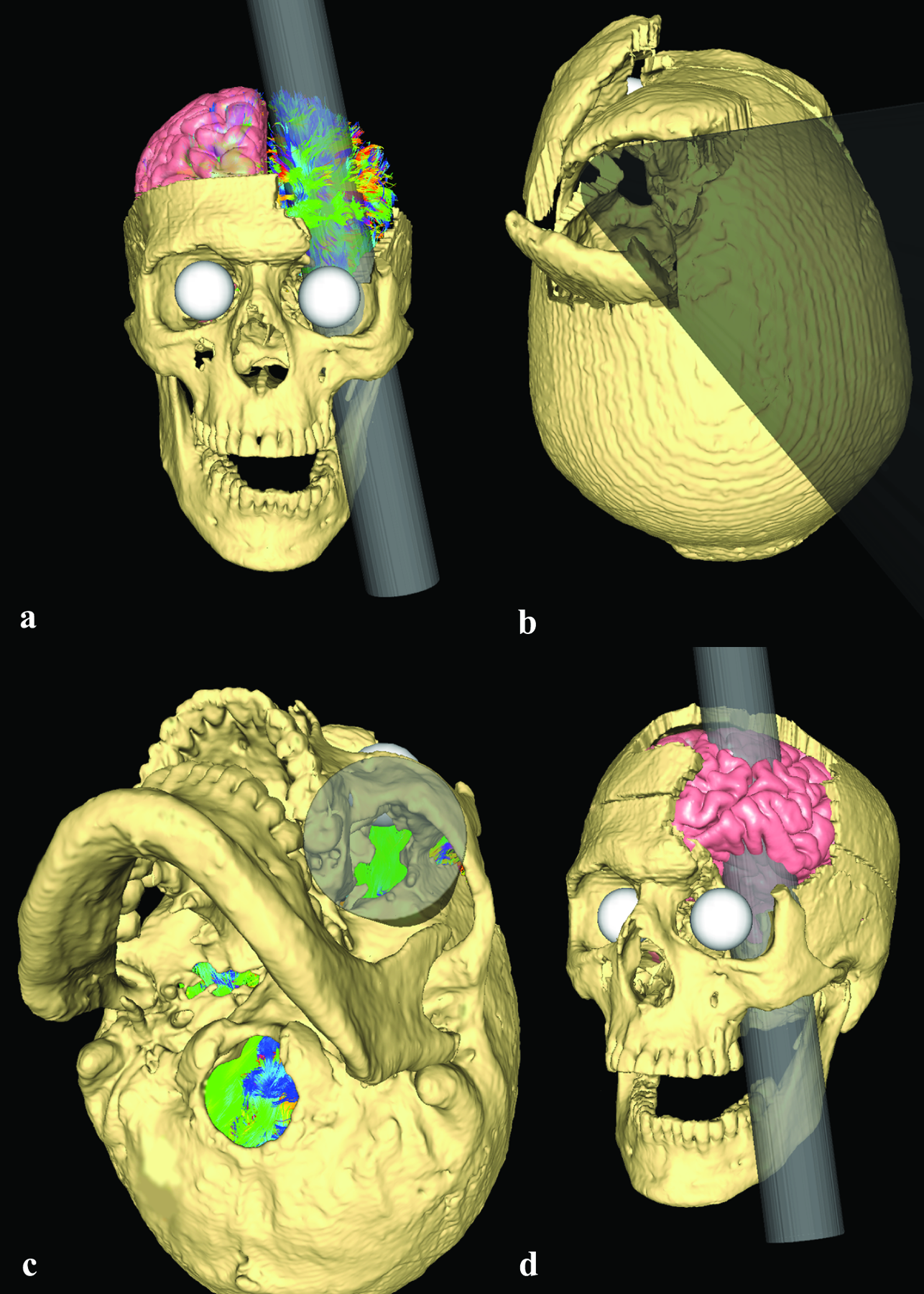

Supplement: Figure S5 — Illustrating the Intersection of the Rod and the Brain. a) A figure showing the passage of the rod through the skull with the bones above the cranial “cap” cut at Harlow's direction, and its intersection with the left anterior white matter fiber pathways of an example subject. The complementary hemisphere is displayed to illustrate that the rod did not intersect that hemisphere. b) A view of the rod displacing the bones of the skull. c) A close up, coxial view of the inferior portion of the iron along its trajectory. d) The intersection of the tamping iron with the left frontal cortex with each major bone fragment removed. (TIF) [file pone.0037454.s005.tif]

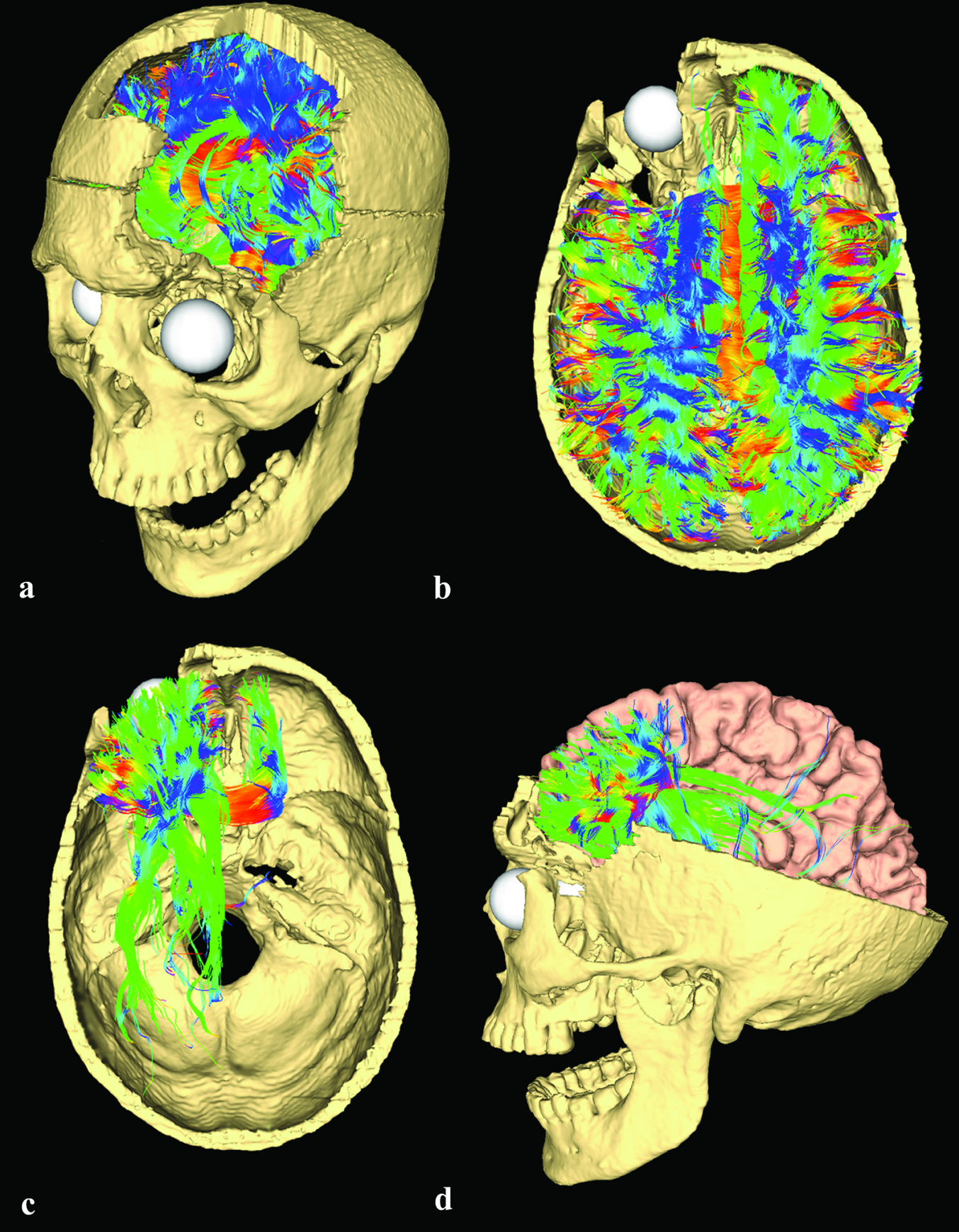

Supplement: Figure S6 — The Effects of the Tamping Iron on White Matter Fiber Tractography. a) A view of the Gage skull with the white matter fiber tracts of an example subject warped to the space. In this view, fibers which intersect the rod's pathway have been removed. b) A transaxial view of the DTI fiber pathways remaining after those which were intersected by the rod had been removed. c) The fibers intersected by the rod connect areas of cortex throughout the left cerebral hemisphere as well as between hemispheres. d) A sagittal view of the fibers experiencing damage by the tamping iron. All bone fragments and the cranial “cap” have been removed. (TIF) [file pone.0037454.s006.tif]
